# Influence of Genetics on the Response to Omalizumab in Patients with Severe Uncontrolled Asthma with an Allergic Phenotype

**DOI:** 10.3390/ijms24087029

**Published:** 2023-04-10

**Authors:** Susana Rojo-Tolosa, José Antonio Sánchez-Martínez, Laura Elena Pineda-Lancheros, José María Gálvez-Navas, María Victoria González-Gutiérrez, Gonzalo Jiménez-Gálvez, Cristina Pérez-Ramírez, Concepción Morales-García, Alberto Jiménez-Morales

**Affiliations:** 1 Respiratory Medicine Department, University Hospital Virgen de las Nieves, 18014 Granada, Spain; susanarojotolosa@gmail.com (S.R.-T.);; 2 Pharmacy Service, Pharmacogenetics Unit, University Hospital Virgen de las Nieves, 18014 Granada, Spain; 3 Center of Biomedical Research, Department of Biochemistry and Molecular Biology II, Institute of Nutrition and Food Technology “José Mataix”, University of Granada, Avda. del Conocimiento s/n., 18016 Granada, Spain; 4 Cancer Registry of Granada, Andalusian School of Public Health, Carretera del Observatorio, 4, 18011 Granada, Spain

**Keywords:** severe uncontrolled asthma, omalizumab, effectiveness, polymorphisms

## Abstract

Omalizumab is a monoclonal antibody indicated for the treatment of severe uncontrolled asthma with an allergic phenotype. Its effectiveness could be influenced by clinical variables and single nucleotide polymorphisms (SNPs) in one or more of the genes involved in the mechanism of action and process of response to omalizumab, and these could be used as predictive biomarkers of response. We conducted an observational retrospective cohort study that included patients with severe uncontrolled allergic asthma treated with omalizumab in a tertiary hospital. Satisfactory response after 12 months of treatment was defined as (1) Reduction ≥ 50% of exacerbations or no exacerbations, (2) Improvement of lung function ≥ 10% FEV1, and (3) Reduction ≥ 50% of OCS courses or no OCS. Polymorphisms in the FCER1A (rs2251746, rs2427837), FCER1B (rs1441586, rs573790, rs1054485, rs569108), C3 (rs2230199), FCGR2A (rs1801274), FCGR2B (rs3219018, rs1050501), FCGR3A (rs10127939, rs396991), IL1RL1 (rs1420101, rs17026974, rs1921622), and GATA2 (rs4857855) genes were analyzed by real-time polymerase chain reaction (PCR) using TaqMan probes. A total of 110 patients under treatment with omalizumab were recruited. After 12 months of treatment, the variables associated with a reduction in exacerbations were the absence of polyposis (odds ratio [OR] = 4.22; 95% confidence interval [CI] = 0.95–19.63), IL1RL1 rs17026974-AG (OR = 19.07; 95% CI = 1.27–547), and IL1RL1 rs17026974-GG (OR = 16.76; 95% CI = 1.22–438.76). Reduction in oral corticosteroids (OCS) was associated with age of starting omalizumab treatment (OR = 0.95; 95% CI = 0.91–0.99) and blood eosinophil levels > 300 cells/µL (OR = 2.93; 95% CI = 1.01–9.29). Improved lung function showed a relationship to the absence of chronic obstructive pulmonary disease (COPD) (OR = 12.16; 95% CI = 2.45–79.49), FCGR2B rs3219018-C (OR = 8.6; 95% CI = 1.12–117.15), GATA2 rs4857855-T (OR = 15.98; 95% CI = 1.52–519.57) and FCGR2A rs1801274-G (OR = 13.75; 95% CI = 2.14–142.68; AG vs. AA and OR = 7.46; 95% CI = 0.94–89.12; GG vs. AA). Meeting one response criterion was related to FCER1A rs2251746-TT (OR = 24; 95% CI = 0.77–804.57), meeting two to age of asthma diagnosis (OR = 0.93; 95% CI = 0.88–0.99), and meeting all three to body mass index (BMI) < 25 (OR = 14.23; 95% CI = 3.31–100.77) and C3 rs2230199-C (OR = 3; 95% CI = 1.01–9.92). The results of this study show the possible influence of the polymorphisms studied on the response to omalizumab and the clinical benefit that could be obtained by defining predictive biomarkers of treatment response.

## 1. Introduction

Asthma is a chronic inflammatory disease involving bronchial hyperreactivity and variable, totally or partially reversible obstruction of airflow, which may give rise to episodes of dyspnea, wheezing, and exacerbations or attacks [1]. Asthma is a problem of worldwide significance, being one of the most serious medical conditions affecting all age groups. World Health Organization (WHO) estimates reveal that 235 million people are affected by this disease, and nearly 2.5 million die every year [2].

Asthma is one of the most important pulmonary diseases. It entails a significant global burden on healthcare systems and society, owing to the high costs it generates and the loss of quality of life and work productivity of patients suffering from moderate to severe disease [3].

The main challenge for asthma treatment lies in its diversity, which is mediated to some degree by environmental and genetic factors. Given the complexity of the disease and the difficulty of controlling it, the most promising options have emerged in the last decade with the development of biological therapies as an alternative and/or supplement to treatment, specifically monoclonal antibodies, which treat the underlying inflammation by blocking selected targets [4].

Omalizumab (marketed as Xolair) is a humanized monoclonal antibody (IgG1 kappa) that binds selectively to immunoglobulin E (IgE) and is indicated for the treatment of severe allergic asthma [5]. The molecular structure of IgE includes two variable fragments (Fab), which interact with specific antigens, and a constant region (Fc) that binds to IgE receptors. The IgE molecule is composed of two identical light chains, which have a variable and a constant part, paired with two identical heavy chains, with a variable portion consisting of a unique domain and a constant fragment that includes four domains (Cε1, Cε2, Cε3, Cε4) [6]. In allergic asthma, the pathogenic role of IgE depends on its binding, through the Cε3 domains, to high-affinity (FcεRI) and low-affinity (FcεRII/CD23) receptors expressed by many cells. Omalizumab binds selectively to the Cε3 domains, including the binding sites both for FcεRI and for FcεRII/CD23, preventing IgE from binding to the high- and low-affinity receptors [6]. Consequently, omalizumab inhibits all the cellular processes dependent on the interaction of IgE with its receptors: mast cell degranulation, basophil expression of high-affinity IgE receptors, eosinophil survival, presentation of antigens to T cells, and IgE synthesis [7,8].

The function of the Fc region of immunoglobulin G (IgG) is to improve the drug’s pharmacokinetics, increasing its stability and prolonging its half-life [9]. The Fc region of IgG1 binds selectively to the Fc-gamma receptors (FcγR), which are integral membrane glycoproteins that exhibit complex activation or inhibitory effects on antibody cell functions after aggregation by IgG [10,11]. There are various types of FcγR: FcγRI (CD64), FcγRII (CD32), and FcγRIII (CD16), in which the affinity for the Fc region of IgG can vary, modifying the response to biological therapies (BTs) [10]. In addition, the affinity of these receptors for the Fc region of IgG1 of omalizumab could vary due to structural changes in the extracellular domain of the receptors, interfering with the therapeutic response.

The interleukin 1 receptor-like 1 gene (IL1RL1) has been considered in numerous genome-wide association studies (GWASs) as a gene for asthma susceptibility, response to inhaled corticosteroids (ICS), and the significant increase in serum IgE [12,13,14,15,16], and could affect the response to omalizumab.

GATA-binding factor 2 (GATA2), a member of the GATA family of transcription factors, plays an essential role in the differentiation of mast cells. These present FcεRI on the cell surface, which binds selectively to IgE, triggering the allergic reaction [17]. GATA2 binds to the promoter region of FCER1A (which encodes FcεRIα) and to the promoter of FCER1B (which encodes FcεRIβ), regulating FcεRI expression and IgE-mediated degranulation activity [18].

Within this conceptual framework, the objective of this study was to assess the participation of SNPs, as the most common cause of disease-associated genetic polymorphisms [19], in some of the genes involved in the mechanism of action and the process of response to omalizumab (FCER, FCGR, C3, IL1RL1, GATA2), to obtain predictive biomarkers of response to this biological therapy.

## 2. Results

### 2.1. Characteristics of the Patients

A total of 74 patients treated with omalizumab were included. The clinical and socio-demographic data are shown in Table 1. The mean age of all the patients was 46.89 ± 16.74 years, and 65.33% (48/74) were women. A total of 76.71% (56/74) had a BMI greater than 25, 21.33% (16/74) were ex-smokers, and 4% (3/74) were active smokers. During the year before starting omalizumab, the median number of ICS doses was 500 (250,1000), 74.67% (56/74) had received at least one course of OCS, 59.15% (56/74) had %FEV1 of less than 80%, 64% (48/74) had suffered at least one exacerbation requiring emergency department treatment and/or hospitalization, and median blood IgE was 359 (151,980) IU/mL. The remaining socio-demographic and clinical variables are described in Table 1.

### 2.2. Clinical Effectiveness of Omalizumab

The effectiveness of omalizumab was evaluated 12 months after starting omalizumab treatment. After 12 months, 95.95% (71/74) showed a satisfactory response in respect of at least one criterion, 85.15% (63/74) in respect of at least two criteria, and 45.59% (31/74) in respect of all three response criteria. Furthermore, 86.49% (64/74) responded satisfactorily with respect to a reduction in exacerbations, 76.47% (52/74) with respect to improvement in lung function, and 66.22% (49/74) with respect to a reduction in OCS (Table 2).

### 2.3. Distribution of the Genotypes Analyzed

**Figure 1:**
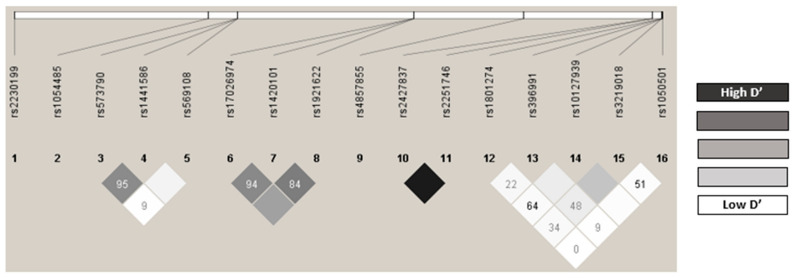
Linkage disequilibrium (LD).

The observed genotype frequencies coincided with the expected values according to the Hardy–Weinberg equilibrium (HWE) model, except for IL1RL1 rs1921622 (*p* = 0.010, Table S1). No statistical differences from those described in Iberian populations were found for this variant (IL1RL1 rs1921622 A allele: 0.649 vs. 0.477; *p* = 0.806) [20]. The D′ and r2 LD values are shown in Table S2, and Figure 1 shows the LD graph. The following pairs of polymorphisms were in strong linkage disequilibrium: IL1RL1 rs17026974/rs1420101 (D′ = 0.94), IL1RL1 rs1420101/rs1921622 (D′ = 0.84), FCER1B rs573790/rs1441586 (D′ = 0.95), and FCER1A rs2427837/rs2251746 (D′ = 1) (Table S2, Figure 1). All the polymorphisms showed a minor allele frequency (MAF) of more than 1%; therefore, none were excluded for analysis (Table S3). The estimated haplotype frequencies are presented in Tables S4–S9.

### 2.4. Predictors of Omalizumab Response at 12 Months

#### 2.4.1. Predictors of Response for Exacerbation Reduction

In the bivariate analysis, the variables associated with a satisfactory response in respect of reduction in exacerbations were the absence of polyposis (OR = 3.92; 95% CI = 0.96–16.19; *p* = 0.042) and SAHS (OR = 4.15; 95% CI = 1.06–17.97; *p* = 0.034) (Table S10). With regard to the pharmacogenetic variables, an association was found between satisfactory response and the FCER1B rs573790-T allele (OR = 3.92; 95% CI = 0.96–16.19; *p* = 0.042), the C3 rs2230199-C allele (OR = 3.92; 95% CI = 0.96–16.19; *p* = 0.042), and the IL1RL1 rs17026974-G allele for the genotypic and recessive models (OR = 16.67; 95% CI = 1.25–433.96; *p* = 0.06; AG vs. AA, OR = 15.2; 95% CI = 1.25–365.47; *p* = 0.06; GG vs. AA, and OR = 15.75; 95% CI = 1.36–362.71; *p* = 0.046; G vs. AA) (Table S11).

The multivariate analysis showed that the independent variables associated with a satisfactory response for reduction in exacerbations after 12 months of omalizumab treatment were the absence of polyposis (OR = 4.22; 95% CI = 0.95–19.63), the IL1RL1 rs17026974-AG genotype (OR = 19.07; 95% CI = 1.27–547; AG vs. AA), and the IL1RL1 rs17026974-GG genotype (OR = 16.76; 95% CI = 1.22–438.76; GG vs. AA) (Table 3).

#### 2.4.2. Predictors of Response for OCS Reduction

In the bivariate analysis, the variables associated with a satisfactory response in respect of reduction in OCS were age of starting omalizumab treatment (OR = 0.96; 95% CI = 0.93–1; *p* = 0.039), patients with BMI < 25 (OR = 9.6 × 10^8^; 95% CI = 5.7 × 10^−35^–NA; *p* < 0.001), age of asthma diagnosis (OR = 0.96; 95% CI = 0.92–0.99; *p* = 0.016), and %FEV1 values of >80% and blood eosinophils of >300 cells/µL during the year prior to receiving omalizumab (OR = 2.88; 95% CI = 1.01–9.11; *p* = 0.052 and OR = 2.57; 95% CI = 0.93–7.54; *p* = 0.071, respectively) (Table S12). No association was found between a satisfactory response and any genetic variants (Table S13).

The multivariate analysis revealed that the independent variables associated with a satisfactory response in respect of reduction in OCS after 12 months of omalizumab treatment were age of starting omalizumab (OR = 0.95; 95% CI = 0.0.91–0.99) and blood eosinophil levels > 300 cell/µL (OR = 2.93; 95% CI = 1.01–9.29) (Table 3).

#### 2.4.3. Predictors of Response for Lung Function Improvement

In the bivariate analysis, the variable associated with a satisfactory response in respect of improvement in lung function were age of starting omalizumab (OR = 0.94; 95% CI = 0.9–0.98; *p* = 0.018), BMI < 25 (OR = 5.68; 95% CI = 1–107.39; *p* = 0.076), absence of previous respiratory disease (OR = 6.14; 95% CI = 1.85–21.81; *p* = 0.002), absence of COPD (OR = 15.43; 95% CI = 3.98–71.29; *p* < 0.001), and age of asthma diagnosis (OR = 0.95; 95% CI = 0.91–0.99; *p* = 0.019) (Table S14). As regards the pharmacogenetic variables, an association and/or tendency was found between satisfactory response and the FCGR2A rs1801274-G allele for the genotypic and recessive models (OR = 5.25; 95% CI = 1.42–22.64; *p* = 0.052; AG vs. AA, OR = 3; 95% CI = 0.7–16.13; *p* = 0.052; GG vs. AA, and OR = 4.29; 95% CI = 1.33–14.49; *p* = 0.012; G vs. AA), the FCGR2B rs3219018-C allele (OR = 4.37; 95% CI = 1.07–29.76; *p* = 0.052), the FCGR3A rs396991-C allele (OR = 4.22; 95% CI = 1.18–17.63; *p* = 0.072; CA vs. AA y OR = 1.13; 95% CI = 0.23–6.35; *p* = 0.072; CC vs. AA) and the GATA2 rs4857855-T allele (OR = 6.08; 95% CI = 1.07–114.8; *p* = 0.062) (Table S15).

The multivariate analysis showed that the independent variables associated with a satisfactory response in respect of improvement in lung function after 12 months of treatment with omalizumab were the absence of COPD (OR = 12.16; 95% CI = 2.45–79.49), the FCGR2B rs3219018-C allele (OR = 8.6; 95% CI = 1.12–117.15), the GATA2 rs4857855-T allele (OR = 15.98; 95% CI = 1.52–519.57), and the FCGR2A rs1801274-G allele for the genotypic model (OR = 13.75; 95% CI = 2.14–142.68; AG vs. AA, and OR = 7.46; 95% CI = 0.94–89.12; GG vs. AA, respectively) (Table 3).

#### 2.4.4. Predictors of Meeting at Least One Response Criterion

In the bivariate analysis, the variables associated with meeting at least one response criterion satisfactorily were age of starting omalizumab (OR = 0.87; 95% CI = 0.73–0.98; *p* = 0.061), absence of GERD (OR = 6.3 × 10^8^; 95% CI = 1.18 × 10^−212^–NA; *p* = 0.006), and age of asthma diagnosis (OR = 0.86; 95% CI = 0.72–0.97; *p* = 0.036) (Table S16). With regard to the pharmacogenetic variables, a tendency was found between a satisfactory response and the FCER1A rs2251746-T allele for the genotypic and recessive models (OR = 10.5; 95% CI = 0.33–356.19; *p* = 0.088; CT vs. CC, OR = 24; 95% CI = 0.77–804.57; *p* = 0.088; TT vs. CC, and OR = 17.25; 95% CI = 0.65–291.88; *p* = 0.008; G vs. AA) (Table S17).

In the multivariate analysis, the independent variable that maintained an association with meeting at least one response criterion satisfactorily after 12 months of omalizumab treatment was the FCER1A rs2251746-TT genotype (OR = 24; 95% CI = 0.77–804.57) (Table 3).

#### 2.4.5. Predictors of Meeting at Least Two Response Criteria

In the bivariate analysis, the variables associated with meeting at least two response criteria satisfactorily were age of starting omalizumab (OR = 0.94; 95% CI = 0.88–0.99; *p* = 0.026), BMI < 25 (OR = 2.8 × 10^6^; 95% CI = 9.88 × 10^−46^–NA; *p* = 0.045), and age of asthma diagnosis (OR = 0.93; 95% CI = 0.88–0.99; *p* = 0.018) (Table S18). Regarding the pharmacogenetic variables, an association was found between a satisfactory response and the GATA2 rs4857855-T allele (OR = 7.8 × 10^7^; 95% CI = 1.18 × 10^−74^–NA; *p* = 0.35; T vs. CC) (Table S19).

In the multivariate analysis, the independent variable that maintained an association with meeting at least two criteria satisfactorily after 12 months of omalizumab treatment was age of asthma diagnosis (OR = 0.93; 95% CI = 0.88–0.99) (Table 3).

#### 2.4.6. Predictors of Meeting All Three Criteria

In the bivariate analysis, the variables associated with meeting the three response criteria were age of starting omalizumab (OR = 0.96; 95% CI = 0.92–0.99; *p* = 0.01), BMI < 25 (OR = 13.38; 95% CI = 3.23–92.3; *p* < 0.001), absence of previous respiratory disease (OR = 4.11; 95% CI = 1.27–16.1; *p* = 0.02), absence of polyposis (OR = 5.05; 95% CI = 1.43–23.94; *p* = 0.014), absence of GERD (OR = 3.46; 95% CI = 0.9416.67; *p* = 0.069), absence of SAHS (OR = 5.74; 95% CI = 1.8–22.36; *p* = 0.003), absence of COPD (OR = 5.05; 95% CI = 1.43–23.98; *p* = 0.014), age of asthma diagnosis (OR = 0.953; 95% CI = 0.92–0.98; *p* = 0.006), and previous %FEV1 < 80% (OR = 3.43; 95% CI = 1.24–10.06; *p* = 0.069) (Table S20). With regard to the pharmacogenetic variables, an association was found between satisfactory response and the C3 rs2230199-C allele for the dominant model (OR = 2.91; 95% CI = 1.06–8.43; *p* = 0.039; C vs. GG) and a tendency for the genotypic model (OR = 1.75; 95% CI = 0.07–43.31; *p* = 0.077; CC vs. GG and OR = 3.06; 95% CI = 1.07–9.22; *p* = 0.077; CG vs. GG) (Table S21).

The multivariate analysis revealed that the independent variables associated with a satisfactory response for all three criteria after 12 months of omalizumab treatment were BMI < 25 (OR = 14.23; 95% CI = 3.31–100.77) and the C3 rs2230199-C allele (OR = 3; 95% CI = 1.01–9.92) (Table 3).

## 3. Discussion

The response in patients diagnosed with severe uncontrolled asthma with an allergic phenotype is variable [21,22,23,24]. The search for predictive biomarkers of a predisposition to asthma, exacerbations, and response to treatment with ICS or short-action β2-adrenergic receptor agonists (SABAs) has been the main objective of numerous research studies over the past few years. Omalizumab has already proved its effectiveness in reducing symptoms, using rescue medication, as well as improving patients’ quality of life in many controlled randomized clinical trials [25,26,27,28,29,30,31,32,33,34,35,36] and real-life studies [6,37,38,39,40,41]. Various authors endorse the major genetic contribution to predisposition to asthma, with estimates of up to 74% in adults and 90% in children [42,43,44,45,46,47]. There have also been studies on the role of pharmacogenetics in response to treatment with inhaled SABAs [48] and ICS [49,50,51]. However, no studies report genetic biomarkers associated with the response to BTs, such as omalizumab, in asthmatic patients. For this purpose, we need to evaluate the effectiveness of treatments in different populations and find the biomarkers that determine that effectiveness. In our study, after 12 months of treatment with omalizumab, the highest proportion of responders consisted of those who showed a satisfactory improvement in at least one of the response criteria (95.6%), followed by those who obtained a reduction of 50% or more in the rate of exacerbations (86.5%), those who met at least two response criteria (85.1%), those who obtained an improvement of 10% or more in %FEV1 or %FEV1 of 80% or more (76.5%), those who had a reduction of 50% or more in OCS (66.2%), and those who met all three response criteria (45.6%). A study was carried out by Casale et al. on 806 patients (United States) with asthma. It showed that after 12 months of treatment with omalizumab, 77.8% responded with an exacerbation reduction of 50% or more, in line with our results, 35.9% with an improvement of 10% or more in FEV1, somewhat lower than that obtained in our study, and 64.7% improved their Asthma Control Test (ACT) score, a variable that we could not use owing to the lack of prior data. In agreement with our result, they found that 86.9% met at least one of the response criteria [52]. In our study, the response biomarkers found were older age of starting BT and diagnosis of the disease, associated with a lower probability of response in the improvement of lung function, absence of previous pulmonary disease, and BMI of less than 25, with a higher probability of pulmonary improvement. Prior %FEV1 values greater than 80%, a blood eosinophil count of >300 cells/mL, and BMI results of <25 were related to a higher probability of a reduction in courses of OCS; absence of polyposis and GERD, with a better response for reduction of exacerbations requiring emergency treatment. Several authors report, in line with our results, that eosinophil levels of >300 cells/mL mean a better response to omalizumab and fewer exacerbations, consequently involving a reduction in courses of OCS [53,54,55]. Casale et al. found a relationship between improvement in lung function and a high eosinophil level (*p* = 0.011) and a higher risk of exacerbations if they had been present the previous year (OR = 2.19; 95% CI = 1.55–3.08; *p* < 0.001); however, they did not seek biomarkers associated with the OCS-saving effect [52]. Other authors who assessed the overall response found a relationship between high IgE and blood eosinophil values and lower %FEV1 levels with a greater response to omalizumab [38,40,41,56].

Among the results reported, several biomarkers have been identified as possible predictors of response to omalizumab. Pharmacogenetics could be the perfect complementary tool to take maximum advantage of the information we already have and bring us closer to achieving personalized medicine, which could optimize the treatment of patients by enabling us to know in advance the response/toxicity that they could present. In addition, polymorphisms in some of the genes involved in the mechanism of action of omalizumab, such as FCER1, C3, IL1RL1, and GATA2, could play a part as a supplement to these response biomarkers. So far, no study has investigated the participation of these SNPs or others in the therapeutic response to omalizumab.

The gene that codes for the high-affinity IgE receptor alpha chain (FCER1A) is located on chromosome 1q23. This gene has two SNPs, rs2251746 (T > C) and rs2427837 (C > T), that have been extensively studied because of their association with high serum IgE levels [57,58,59]. Our study found that for the FCER1A rs2251746 SNP, carriers of the CT and TT genotypes and the T allele showed a higher probability of meeting at least one response criterion than those with the CC genotype. Its association with omalizumab response has not been studied before. Still, Liao et al. related the CT and TT genotypes of the rs2251746 SNP with higher levels of total IgE, specific IgE, and IgE-secretion B cells (*p* < 0.001) [57]. These results are endorsed by other authors [58,59]. The association of this SNP with a genetic predisposition to higher IgE levels and a greater response to omalizumab is in line with the previously reported biomarkers [38]. No associations were found between these SNPs and the other responses evaluated.

The beta subunit of the high-affinity immunoglobulin epsilon receptor is a protein encoded in humans by the FCER1B gene (also known as MS4A2), which is located on the 11q12-13 chromosome. This gene includes the rs1441586 (T > C), rs573790 (T > C), rs1054485 (T > G), and rs569108 (A > G) SNPs, common in asthmatic pathology and atopy and related to high IgE levels [60,61,62,63,64]. In our study, we only found a significant association between the FCER1B rs573790-CC genotype and a higher probability of response in respect of a reduction in exacerbations compared to the T allele. There have been no reports of studies of an association of this SNP with response to omalizumab. Still, the presence of the rs573790-CC genotype has been reported in patients with aspirin-exacerbated respiratory disease (AERD) and asthma [65].

The C3 rs2230199 (G > C) polymorphism produces a change from arginine to glycine (p.Arg102Gly), giving rise to a missense variant that partially alters the functionality of complement in asthma [66]. In our study, we found an association between the C3 rs2230199-C allele and a higher probability of response in respect of the reduction of exacerbations with the use of omalizumab. Furthermore, the C3 rs2230199-CC and CG genotypes were also linked to a greater predisposition to meeting all three criteria. The relationship of this SNP to the response to omalizumab has not been studied before. Still, there are authors that associate changes in the gene’s functionality with alterations in interleukin-4 (IL4) production and specific IgE and IgG responses [66] which leads us to think that if the functionality of the gene is altered, the binding of omalizumab to IgE could also be altered.

In the FcγR receptors, there have been studies of SNPs in the FCGR2A, FCGR2B, and FCGR3A genes, located on chromosome 1q23.3, that could affect the stability of the bond in the Fc region. Our results only reported significant associations of SNPs in these genes to the response of improvement in lung function. The FCGR2A rs1801274-G, FCGR2B rs3219018-C, and FCGR3A rs396991-C alleles were linked to a greater response. The FCGR2A rs1801274 (A > G) SNP gives rise to a replacement of histidine (His) by arginine (Arg) (His131Arg) [67,68]. Previous studies on other diseases have shown that FCGR2A with His instead of Arg at position 131 resulted in a higher affinity for IgG1 [69], which may explain the association of the G allele with response. Another study in an asthmatic population showed that the FCGR2A rs1801274-G allele was significantly associated with atopy, while the A allele had a protective role [70]. The FcγRIIb receptor is an inhibitory IgG receptor whose SNPs correlate with negative regulation of FcγRIIbin in B cells and positive regulation of IgG antibody responses [71,72]. This could explain the association of the C allele of the FCGR2B rs3219018 (G > C) SNP with a better response, corresponding in this case to positive regulation of IgG. The FCGR3A rs396991 (A > C) SNP produces a replacement of phenylalanine (Phe) by valine (Val) (Phe158Val). Previous studies with other BTs show that the low-affinity variant FCGR3A-p.158Phe is associated with lesser clearance of the BT and, consequently, with a better therapeutic response [69].

The IL1RL1 gene is on chromosome 2q12. Previous studies indicate that the SNPs studied (rs1420101, rs17026974, rs1921622) could act as promoters of type 2 inflammatory response in the airways, with higher serum IgE counts, high eosinophilia, and lesser FEV1 reversibility; however, their relationship to BT response has not been studied [12,13,73]. Our study only found a positive association between the IL1RL1 rs17026974-G allele and the reduction of exacerbations.

The GATA2 gene is located on chromosome 3q21. Our results report a significant association of the GATA2 rs4857855-T allele with improved lung function and meeting at least two parameters. Previous research underlines the important role of GATA2 in developing hematopoietic and Th2 cells, associating this SNP with high eosinophil counts [13,74]. Still, its role in response to BTs has not been studied.

The main limitation of this study was the sample size, which may be responsible for the absence of a statistically significant association between some of the genetic variables and the types of responses analyzed. To ensure the uniformity and reliability of the variables collected, all the patients were recruited from the same hospital cohort, with the same procedures, and through the same staff. Another inherent limitation of a retrospective study is the lack of some clinical data of interest for some patients. Nevertheless, the effects observed were clear, although studies are required to assess the prognostic value of these polymorphisms in larger cohorts.

## 4. Material and Methods

### 4.1. Study Design

We conducted an observational retrospective cohort study.

### 4.2. Study Population

This study included 110 patients aged over 18 years and of Caucasian origin diagnosed with severe uncontrolled asthma according to the criteria of the Spanish Asthma Management Guidelines (GEMA 5.2) [1], recruited in the Respiratory Medicine Department of the Hospital Universitario Virgen de las Nieves in Granada (Spain) between March 2007 and October 2022. Out of the 110 patients recruited, the response to omalizumab was evaluated in 74 prior to beginning treatment and 12 months after starting biological therapy. The remaining patients did not meet the study’s evaluation criteria. The administration route of the drug was subcutaneous, with doses of 75 mg to 600 mg of omalizumab, depending on the initial IgE concentration and the patient’s weight, every 2 or 4 weeks [75].

### 4.3. Ethics Statements

The study was carried out with the approval of the Ethics and Research Committee of the Hospital Universitario Virgen de las Nieves (HUVN) in accordance with the Declaration of Helsinki. The subjects who participated in the study signed an informed consent form for collection and genetic analysis of saliva samples and for their donation to the Biobank of the Sistema Sanitario Público de Andalucía (Andalusian Public Health Service). The samples were identified by alphanumeric codes.

### 4.4. Socio-Demographic and Clinical Variables

The socio-demographic and clinical data were collected by reviewing clinical histories. The socio-demographic data collected were age, sex, body mass index (BMI), smoking status, years with the disease, nasal polyposis, previous respiratory disease, allergies, gastroesophageal reflux disease (GERD), sleep apnea-hypopnea syndrome (SAHS), chronic obstructive pulmonary disease (COPD), years of BT, treatment dose, and change to another BT. The clinical variables included courses of oral corticosteroids (OCS) expressed as prednisone-equivalent mg and of ICS expressed as fluticasone-furoate-equivalent µg, blood eosinophil count, exacerbations requiring emergency department treatment and/or hospitalization, IgE, lung function as maximum percentage expiratory volume in the first second of forced exhalation (%FEV1), and Asthma Control Test (ACT) results [76]. The clinical variables were collected with reference to the year before starting BT and after completing the first year of treatment.

### 4.5. Genetic Variables

#### 4.5.1. DNA Isolation

The saliva samples were collected in BD Falcon 50 mL conical tubes (BD, Plymouth, UK). The DNA was extracted using the QlAamp DNA Mini Kit (Qiagen GmbH, Hilden, Germany), following the manufacturer’s instructions for purifying DNA from saliva, and was stored at −40 °C. The concentration and purity of the DNA were measured using a NanoDrop 2000 UV spectrophotometer with 280/260 and 280/230 absorbance ratios.

#### 4.5.2. Detection of Gene Polymorphisms and Quality Control

The gene polymorphisms were determined by real-time polymerase chain reaction (PCR) allelic discrimination assay using TaqMan probes (ABI Applied Biosystems, QuantStudio 3 Real-Time PCR System, 96 wells) according to the manufacturer’s instructions (Table 4). This procedure is based on fluorescent oligonucleotide probes labeled with a fluorescent reporter and a quencher; the two are tightly coupled if the probe does not hybridize to its target sequence, so there is no amplification and no fluorescence signal. When it hybridizes, conformational changes occur in the reporter and quencher, allowing the 5′-3′ exonuclease activity of Taq polymerase to break this bond, allowing the fluorescence emitted by the reporter to be released and captured by the device. Each allele is labeled with a different fluorochrome so that the genotype is determined according to the fluorescence captured by the device. The FCGR2B rs3219018 and rs1050501 polymorphisms were analyzed using a custom assay by ThermoFisher Scientific (Waltham, Massachusetts, United States) coded as ANPRZAZ and ANRWUVX, respectively. Ten percent of the results were confirmed by Sanger sequencing. Real-time PCR and Sanger sequencing were performed in the Pharmacogenetics Unit of the Hospital Universitario Virgen de las Nieves. The criteria for SNP quality control were (1) missing genotype rate per SNP < 0.05; (2) minor allele frequency > 0.01; (3) *p*-value > 0.05 in Hardy–Weinberg equilibrium test; (4) missing genotype rate between cases and control < 0.05.

### 4.6. Response Variables

To evaluate the predictors of response at 12 months, the following were taken as response variables: reduction of OCS courses per year, considering a reduction of at least 50% in courses or absence of OCS as a satisfactory response; improvement of lung function, considering those that achieved an increase of at least 10% in FEV1 after 12 months’ treatment as responders; and reduction of exacerbations per year requiring emergency department treatment and/or hospitalization, taking a reduction of at least 50% or absence of exacerbations as a satisfactory response. These response variables are based on the suggestions for monoclonal antibody response assessment in the “Consensus Document on Severe Asthma in Adults. Update 2020” [77].

On the other hand, the response to the number of criteria is also evaluated, i.e., those who respond to at least 1 of the above criteria, those who respond to at least 2, and those who respond to all 3 criteria. In this case, it is understood that an individual has a better response the more criteria he/she fulfills. All criteria are evaluated with the same value.

### 4.7. Statistical Analysis

The descriptive analysis was performed with R 4.2.0 software. The quantitative variables were expressed as the mean (±standard deviation) for those that complied with normality and as the median and percentiles (25 and 75) for the variables that did not follow a normal distribution. Normality was confirmed using the Kolmogorov–Smirnov test. The bivariate analysis between the response and the genetic variables was performed with multiple models (genotypic, additive, allelic, dominant, and recessive), using the Pearson χ2 test or applying the Fisher exact test for the qualitative variables. For the quantitative variables, we applied the t-test to the variables that complied with normality and the Mann–Whitney test for non-normal variables. The models were defined as allelic (D vs. d), dominant ((DD, Dd) vs. dd), recessive (DD vs. (Dd, dd), genotypic (DD vs. dd and Dd vs. dd), and additive, where D is the minor allele and d the major allele.

A multivariate (logistic regression) analysis was used to calculate the adjusted odds ratio (OR) and the 95% confidence interval (95% CI) for the possible prognostic factors for response.

The Hardy–Weinberg equilibrium, the haplotype frequency, and the linkage disequilibrium (LD) were determined through Lewontin’s D prime (D′) coefficients and the LD coefficient (r2).

All the tests were 2-sided, with a significance level of *p* < 0.05, and were performed using PLINK free-access software Version 1.9 for whole-genome association analysis [78] and the R 4.2.0 statistical program [79]. The LD was calculated with Haploview 4.2 software [80], and the haplotype analysis was performed with SNPStats [81], a web tool for the analysis of association studies.

## 5. Conclusions

In conclusion, this study highlights polymorphisms in the genes: FCER, FCGR, C3, IL1RL1, and GATA2, which have potential prognostic value for response to omalizumab treatment. However, the functional involvement of most asthma loci is still unknown. More studies are therefore needed to increase our understanding of the impact of these genes and their effect on the response to biological therapies, such as omalizumab, to achieve their use in future clinical practice and guide us towards a more personalized kind of medicine.

## Figures and Tables

**Table 1:** Socio-demographic and clinical characteristics of patients treated with omalizumab.

	N	%	Mean ± SD/ p50 (p25, p75)
Sex			
Women	48	65.33	
Men	26	34.67	
Age of starting BT (years)	74		46.89 ± 16.74
Years with asthma	74		8 (5, 14)
BMI (kg/m^2^)			
<25	17	23.29	
>25	56	76.71	
Previous respiratory disease			
Yes	19	25.33	
No	55	79.67	
Smoking status			
Non-smoker	56	74.67	
Active smoker	3	4	
Ex-smoker	16	21.33	
Polyposis			
Yes	18	24	
No	56	76	
Allergies			
Yes	58	77.3	
No	16	22.67	
GERD			
Yes	14	18.67	
No	61	81.33	
SAHS			
Yes	24	32	
No	51	68	
COPD			
Yes	20	22.67	
No	55	77.33	
Age of diagnosis (years)	74		43 (34, 54)
<18	10	13.33	
>18	65	88.67	
ICS (µg/day)	74		500 (250, 1000)
OCS courses in previous year			
Yes	56	74.67	
No	19	25.33	
Baseline %FEV1			
<80	42	59.15	
>80	29	40.85	
Exacerbations in previous year			
Yes	48	64	
No	27	36	
Baseline blood eosinophils (cells/µL)			
<300	37	54.41	
>300	31	45.59	
Baseline IgE (IU/mL)	65		359 (151, 980)
Years with omalizumab			
<5	52	69.33	
>5	23	30.67	
Change of BT			
Yes	37	49.33	
No	38	50.67	

BMI: body mass index; GERD: gastroesophageal reflux disease; SAHS: sleep apnea-hypopnea syndrome; COPD: chronic obstructive pulmonary disease; ICS: inhaled corticosteroids; OCS: oral corticosteroids; %FEV1: percentage forced expiratory volume in the first second; IgE: immunoglobulin E; BT: biological therapy. Qualitative variables are shown as numbers (percentage, %). Quantitative variables with normal distribution are shown as mean ± standard deviation (SD). Quantitative variables with non-normal distribution are p50 (p25–p75).

**Table 2:** Clinical effectiveness of omalizumab in patients with severe uncontrolled asthma and allergic phenotype.

Response Variable	N	%
Responsive for 1 criterion		
Yes	71	95.95
No	3	4.05
Responsive for 2 criteria		
Yes	63	85.15
No	11	14.86
Responsive for 3 criteria		
Yes	31	45.59
No	37	54.41
Reduction in OCS ≥ 50%		
Yes	49	66.22
No	25	33.78
Reduction in exacerbations ≥ 50%		
Yes	64	86.49
No	10	13.51
Increase in %FEV1 ≥ 10% or %FEV1 ≥ 80%		
Yes	52	76.47
No	16	23.53

OCS: oral corticosteroids; %FEV1: maximum percentage expiratory volume in the first second of forced exhalation.

**Table 3:** Predictors of response after 12 months of omalizumab treatment in patients with severe uncontrolled asthma (multivariate analysis).

	OR (95% CI)	*p*-Value
Response in respect of exacerbation reduction		
Polyposis (No)	4.22 (0.95–19.63)	0.050
*IL1RL1* rs17026974 (AG vs. AA)	19.07 (1.27–547)	0.040
*IL1RL1* rs17026974 (GG vs. AA)	16.76 (1.22–438.76)	0.041
Response in respect of reduction of OCS		
Eosinophils (>300 cll/µL)	2.93 (1.01–9.29)	0.055
Age of starting omalizumab	0.95 (0.91–0.99)	0.032
Response in respect of improved lung function		
COPD (No)	12.16 (2.45–79.49)	0.004
GATA2 rs4857855 (T vs. CC)	15.98 (1.52–519.57)	0.052
FCGR2A rs1801274 (AG vs. AA)	13.75 (2.14–142.68)	0.012
FCGR2A rs1801274 (GG vs. AA)	7.46 (0.94–89.12)	0.076
FCGR2B rs3219018 (C vs. GG)	8.6 (1.12–117.15)	0.052
Meeting at least 1 criterion		
FCER1A rs2251746 (TT vs. CC)	24 (0.77–804.57)	0.045
Meeting at least 2 criteria		
Age of diagnosis	0.93 (0.88–0.99)	0.018
Meeting all 3 criteria		
BMI (<25)	14.23 (3.31–100.77)	0.001
C3 rs2230199 (C vs. GG)	3 (1.01–9.92)	0.063

OR: odds ratio; 95% CI: 95% confidence interval.

**Table 4:** Gene polymorphisms and TaqMan ID.

Gene	SNP	dbSNP ID	Assay ID
*FCER1A* (1q23)	T > C	rs2251746	C___1840470_20
	G > A	rs2427837	C__16233438_20
*FCER1B* (11q12-13)	T > C	rs1441586	C___1842226_10
	T > C	rs573790	C____900105_20
	T > G	rs1054485	C___2932371_10
	A > G	rs569108	C____900116_10
*C3* (19p13.3)	G > C	rs2230199	C__26330755_10
*FCGR2A* (1q23.3)	A > G	rs1801274	C___9077561_20
*FCGR2B* (1q23.3)	G > C	rs3219018	ANPRZAZ
	T > C	rs1050501	ANRWUVX
*FCGR3A* (1q23.3)	A > C	rs10127939	C__57480226_10
	A > C	rs396991	C__25815666_10
*IL1RL1* (2q12)	C > T	rs1420101	C___8906009_20
	G > A	rs17026974	C__33551182_10
	G > A	rs1921622	C___1226146_10
*GATA2* (3q21)	C > T	rs4857855	C__11231076_10

## Data Availability

Not applicable.

## Supplementary Materials

The supporting information can be downloaded at: https://www.mdpi.com/article/10.3390/ijms24087029/s1.
