# Supplementary material for: Influence of Genetics on the Response to Omalizumab in Patients with Severe Uncontrolled Asthma with an Allergic Phenotype
Source: Int J Mol Sci. 2023 Apr 10;24(8):7029. doi: 10.3390/ijms24087029 (PMC10139019; doi:10.3390/ijms24087029)
Supplement: Supplementary file 1 [file ijms-24-07029-s001.zip › Table S1.pdf]

Table S1. Hardy-Weinberg equilibrium for the SNPs included in the study.

CHR	SNP	Gene	Minor allele	Major allele	Genotype counting	Observed heterozygosity	Expected heterozygosity	p-valor
1	rs2427837	FCER1A	A	G	2/21/51	0.2838	0.2808	1
1	rs2251746	FCER1A	C	T	3/22/49	0.2973	0.3068	0.7126
1	rs1801274	FCGR2A	G	A	18/34/22	0.4595	0.4985	0.4916
1	rs396991	FCGR3A	C	A	10/38/26	0.5135	0.4766	0.6279
1	rs10127939	FCGR3A	C	A	01/05/1968	0.06757	0.09012	0.138
1	rs3219018	FCGR2B	C	G	1/24/49	0.3243	0.2896	0.4452
1	rs1050501	FCGR2B	C	T	0/20/54	0.2703	0.2337	0.3411
2	rs17026974	IL1RL1	A	G	3/28/43	0.3784	0.3539	0.7466
2	rs1420101	IL1RL1	T	C	10/34/30	0.4595	0.4635	1
2	rs1921622	IL1RL1	A	G	9/48/17	0.6486	0.4942	0.01018
3	rs4857855	GATA2	T	C	2/17/55	0.2297	0.2435	0.6281
11	rs573790	FCER1B	T	C	9/30/35	0.4054	0.4383	0.5951
11	rs1441586	FCER1B	T	C	15/42/17	0.5676	0.4996	0.3512
11	rs569108	FCER1B	G	A	0/7/67	0.09459	0.09012	1
19	rs2230199	C3	C	G	2/25/47	0.3378	0.3151	0.7221
19	rs1054485	FCER1B	T	G	11/39/24	0.527	0.4846	0.6306

CHR, chromosome; SNP, single nucleotide polymorphism.
