# Supplementary material for: Influence of Genetics on the Response to Omalizumab in Patients with Severe Uncontrolled Asthma with an Allergic Phenotype
Source: Int J Mol Sci. 2023 Apr 10;24(8):7029. doi: 10.3390/ijms24087029 (PMC10139019; doi:10.3390/ijms24087029)
Supplement: Supplementary file 1 [file ijms-24-07029-s001.zip › Table S10.pdf]

Table S10. Association of clinical characteristics of omalizumab-treated patients with reduced and/or no exacerbations.

| Characteristics | N | Response R N(%) | Response NR N(%) | $X^2$ | p-value | Ref. Cat | OR | CI 95% |
|---|---|---|---|---|---|---|---|---|
| Sex | | | | | | | | |
| Female | 48 | 43 (89.6) | 5 (10.4) | 1.121 | 0.289 | | | |
| Male | 26 | 21 (80.8) | 5 (19.2) | | | | | |
| Age of initiation BT (years) | 74 | 64 (86.5) | 10 (13.5) | | 0.126 | | | |
| Years with asthma | 74 | 64 (86.5) | 10 (13.5) | | 0.476 | | | |
| BMI (kg/m2) | | | | | | | | |
| <25 | 17 | 16 (94.1) | 1 (5.9) | 1.1928 | 0.275 | | | |
| >25 | 55 | 46 (83.6) | 9 (16.4) | | | | | |
| Previous respiratory disease | | | | | | | | |
| Yes | 19 | 16 (84.2) | 3 (15.8) | 0.1133 | 0.736 | | | |
| No | 55 | 48 (87.3) | 7 (12.7) | | | | | |
| Tobacco consumption | | | | | | | | |
| Non smoker | 37 | 30 (81.1) | | 1.8164 | 0.472* | | | |
| Current smoker | 3 | 2 (66.7) | 1 (33.3) | | | | | |
| Former smoker | 10 | 9 (90) | 1 (10) | | | | | |
| Polyps | | | | | | | | |
| Yes | 18 | 13 (72.2) | 5 (27.8) | 4.1909 | 0.042 | Si | 3.92 | 0.96-16.19 |
| No | 56 | 51 (91.1) | 5 (8.9) | | | | | |
| Allergies | | | | | | | | |
| Yes | 58 | 49 (84.5) | 9(15.5) | 0.9215 | 0.337 | | | |
| No | 16 | 15 (93.8) | 1 (6.2) | | | | | |
| GERD | | | | | | | | |
| Yes | 14 | 11 (78.6) | 3 (21.4) | 0.9255 | 0.336 | | | |
| No | 60 | 53 (88.3) | 7 (11.7) | | | | | |
| SAHS | | | | | | | | |
| Yes | 23 | 17 (73.9) | 6 (26.1) | 4.5142 | 0.034 | Si | 4.15 | 1.06-17.97 |
| No | 51 | 47 (92.2) | 4 (7.8) | | | | | |
| COPD | | | | | | | | |
| Yes | 19 | 15 (78.9) | 4 (21.1) | 1.2432 | 0.265 | | | |
| No | 55 | 49 (89.1) | 6 (10.9) | | | | | |
| Age of diagnosis (years) | 74 | 64 (86.5) | 10 (13.5) | | 0.074 | | | |
| <18 | 10 | 10 (100) | 0 (0) | 1.8066 | 0.179 | | | |
| >18 | 64 | 54 (84.4) | 10 (15.6) | | | | | |
| ICS (µg/day) | 74 | 64 (86.5) | 10 (13.5) | | 0.846 | | | |
| OCS cycles per year | | | | | | | | |
| Yes | 55 | 48 (87.3) | 7 (12.7) | 0.1133 | 0.736 | | | |
| No | 19 | 16 (84.2) | 3 (15.8) | | | | | |
| Baseline FEV1 (%) | | | | | | | | |
| <80 | 42 | 34 (81) | 8 (19) | 2.0932 | 0.148 | | | |
| >80 | 29 | 27 (93.1) | 2 (6.9) | | | | | |
| Exacerbation in previous year | | | | | | | | |
| Yes | 47 | 41 (87.2) | 6 (12.8) | 0.0616 | 0.804 | | | |
| No | 27 | 23 (85.2) | 4 (14.8) | | | | | |
| Basal blood eosinophils (cell/mcl) | | | | | | | | |
| <300 | 36 | 30 (83.3) | 6 (16.7) | 0.1858 | 0.666 | | | |
| >300 | 31 | 27 (87.1) | 4 (12.9) | | | | | |
| Baseline IgE (IU/Ml) | 65 | 56 (86.2) | 9 (13.8) | | 0.539 | | | |

| Characteristics | N | Response | | $X^2$ | p-value | Ref. Cat | OR | CI 95% |
|---|---|---|---|---|---|---|---|---|
| | | R N(%) | NR N(%) | | | | | |
| Years with Omalizumab | | | | | | | | |
| <5 | 51 | 42 (82.4) | 9 (17.6) | 2.3988 | 0.121 | | | |
| >5 | 23 | 22 (95.7) | 1 (4.3) | | | | | |
| Change of BT | | | | | | | | |
| Yes | 36 | 29 (80.6) | 7 (19.4) | 2.11 | 0.146 | | | |
| No | 38 | 35 (92.1) | 3 (7.9) | | | | | |

BMI, body mass index; GERD, gastroesophageal reflux disease; SAHS, sleep apnea-hypopnea syndrome; COPD, chronic obstructive pulmonary disease; ICS, inhaled corticosteroids; OCS, oral corticosteroids; FEV1, maximum expiratory volume in the first second of forced expiration; IgE, immunoglobulin E; BT, biological therapy.

Ref. Cat, Reference category; NR, Non-Responder; R, Responder; OR, Odds Ratio; CI 95%, Confidence interval; *p-value for Fisher's Exact Test.
