# Supplementary material for: Influence of Genetics on the Response to Omalizumab in Patients with Severe Uncontrolled Asthma with an Allergic Phenotype
Source: Int J Mol Sci. 2023 Apr 10;24(8):7029. doi: 10.3390/ijms24087029 (PMC10139019; doi:10.3390/ijms24087029)
Supplement: Supplementary file 1 [file ijms-24-07029-s001.zip › Table S11.pdf]

Table S11. Association of omalizumab genetic polymorphisms with reduction and/or absence of exacerbations.

Gene	SNPs	Genotype	N	Response R N (%)	Response NR N (%)	χ²	p-value	Ref Cat	OR	CI 95%
IL1RL1	rs1420101	CC	30	26 (86.7)	4 (13.3)					
		CT	34	30 (88.2)	4 (11.8)		0.802*			
		TT	10	8 (80)	2 (20)					
		C	64	56 (87.5)	8 (12.5)	0.4163	0.519			
		T	44	38 (86.4)	6 (13.6)	0.0014	0.970			
	rs17026974	AA	3	1 (33.3)	2 (66.7)					
		AG	28	25 (89.3)	3 (10.7)		0.060*	AA	16.67	1.25-433.96
		GG	43	38 (88.4)	5 (11.6)				15.2	1.25-365.47
		A	31	26 (83.9)	5 (16.1)	0.3123	0.576			
		G	71	63 (88.7)	8 (11.3)		0.046*	AA	15.75	1.36-362.71
	rs1921622	AA	9	7 (77.8)	2 (22.2)					
		AG	48	41 (85.4)	7 (14.6)		0.465*			
		GG	17	16 (94.1)	1 (5.9)					
		A	57	48 (84.2)	9 (15.8)	1.0997	0.294			
		G	65	57 (87.7)	8 (12.3)	0.6649	0.415			
GATA2	rs4857855	CC	55	46 (83.6)	9 (16.4)					
		CT	17	16 (94.1)	1 (5.9)		0.577*			
		TT	2	2 (100)	0 (0)					
		C	72	10 (13.9)	62 (86.1)		1*			
		T	19	18 (94.7)	1 (5.3)		0.437*			
FCER1A	rs2251746	CC	3	2 (66.7)	1 833.3)					
		CT	22	18 (81.8)	4 (18.2)		0.223*			
		TT	49	44 (89.8)	5 (10.2)					
		C	25	20 (80)	5 (20)	1.3592	0.244			
		T	71	62 (87.3)	9 (12.7)		0.357*			
	rs2427837	AA	2	2 (100)	0 (0)					
		AG	21	16 (76.2)	5 (23.38)		0.226*			
		GG	51	46 (90.2)	5 (9.8)					
		A	23	18 (78.3)	5 (21.7)	1.932	0.165			
		G	72	62 (86.1)	10 (13.9)		1*			
FCER1B	rs1441586	CC	17	16 (94.1)	1 (5.9)					
		CT	42	36 (85.7)	6 (14.3)		0.52*			
		TT	15	12 (80)	3 (20)					
		C	59	52 (88.1	7 (11.9)	0.6773	0.411			
		T	57	48 (84.2)	9 (15.8)	1.0997	0.294			
	rs573790	CC	35	33 (94.)	2 (5.7)					
		CT	30	24 (80)	6 (20)		0.135*			
		TT	9	7 (77.8)	2 (22.2)					
		C	65	57 (87.7)	8 (12.3)	0.6649	0.415			
		T	39	31 (79.5)	8 (20.5)	3.4564	0.063	T	4.26	1.01-29.68
	rs1054485	GG	24	22 (91.7)	2 (8.3)					
		GT	39	33 (84.6)	6 (15.4)		0.716*			
		TT	11	9 (81.8)	2 (18.2)					
		G	63	55 (87.3)	8 (12.7)	0.2409	0.624			
		T	50	42 (84)	8 (16)	0.155	0.367			
	rs569108	AA	67	57 (85.1)	10 (14.9)					
		AG	7	7 (100)	0 (0)	1.208	0.272			
		GG	0	0 (0)	0 (0)					
		A	-	-	-					
		G	7	7 (100)	0 (0)	1.208	0.272			
C3	rs2230199	CC	2	2 (100)	0 (0)		0.19*			
		CG	25	24 (96)	1 (4)					
		GG	47	38 (80.9)	9 (19.1)					
		C	27	26 (96.3)	1 (3.7)	3.5003	0.061	GG	6.16	1.06-117.05
		G	72	62 (86.1)	10 (13.9)		1*			
FCGR2A	rs1801274	AA	22	17 (77.3)	5 (22.7)					
		AG	34	31 (91.2)	3 (8.8)		0.345*			
		GG	18	16 (88.9)	2 (11.1)					
		A	56	48 (85.7)	8 (14.3)	0.1175	0.732			
		G	52	47 (90.4)	5 (9.6)	2.2741	0.132			

Gene	SNPs	Genotype	N	Response		χ²	p-value	Ref Cat	OR	CI 95%
				R<br>N (%)	NR<br>N (%)					
FCGR2B	rs3219018	CC	1	1 (100)	0 (0)		0.551*			
		CG	24	22 (91.7)	2 (8.3)					
		GG	49	41 (83.7)	8 (16.3)					
		C	49	41 (83.7)	8 (16.3)	0.9820	0.322			
		G	73	63 (86.3)	110 (13.7)		1*			
	rs1050501	CC	0	0 (0)	0 (0)	0.2895	0.591			
		CT	20	18 (90)	2 (20)					
		TT	54	46 (85.2)	8 (14.8)					
		C	20	18 (90)	2 (20)	0.2895	0.591			
		T	-	-	-					
FCGR3A	rs10127939	AA	68	59 (86.8)	9 (13.2)		0.595*			
		AC	5	4 (80)	1 (20)					
		CC	1	1 (100)	0 (0)					
		A	73	63 (86.3)	10 (13.7)		1*			
		C	68	59 (86.8)	9 (13.2)	0.0555	0.814			
	rs396991	AA	26	23 (88.5)	3 (11.5)		0.361*			
		CA	38	31 (81.6)	7 (18.4)					
		CC	10	10 (100)	0 (0)					
		A	64	54 (84.4)	10 (15.6)	1.8066	0.179			
		C	48	41 (85.4)	7 (14.6)	0.1338	0.715			

Ref. Cat., reference category; R, responder; NR, non-responder; OR, odds ratio; CI 95%, 95% confidence Interval 95%; *p-value for Fisher exact test.
