# Supplementary material for: Influence of Genetics on the Response to Omalizumab in Patients with Severe Uncontrolled Asthma with an Allergic Phenotype
Source: Int J Mol Sci. 2023 Apr 10;24(8):7029. doi: 10.3390/ijms24087029 (PMC10139019; doi:10.3390/ijms24087029)
Supplement: Supplementary file 1 [file ijms-24-07029-s001.zip › Table S12.pdf]

Table S12. Association of clinical characteristics of omalizumab-treated patients with reduction and/or absence of oral corticosteroids.

Characteristics	N	Response<br>R<br>N (%)	 NR<br>N (%)	X^2	p-value	Ref. Cat	OR	CI 95%
Sex								
Female	48	33 (68.8)	15 (31.2)	0.3921	0.531			
Male	26	16 (61.5)	10 (38.5)					
Age of initiation BT (years)	74	49 (66.2)	25 (33.8)		0.039		0.96	0.93-1
Years with asthma	74	49 (66.2)	25 (33.8)		0.986			
BMI (kg/m2)								
<25	17	17 (100)	0 (0)	11.838	<0.001	>25	$9.6e^{8}$	$5.7e^{-35}$-NA
>25	55	30 (54.5)	25 (45.5)					
Previous respiratory disease								
Yes	19	10 (52.6)	9 (47.4)	2.1089	0.146			
No	55	39 (70.9)	16 (29.1)					
Tobacco consumption								
Non smoker	55	36 (65.5)	19 (34.6)	2.3142	0.612*			
Current smoker	3	1 (33.3)	2 (66.7)					
Former smoker	16	12 (75)	4 (25)					
Polyps								
Yes	18	10 (55.6)	8 (44.4)	1.2084	0.272			
No	56	39 (69.6)	17 (30.4)					
Allergies								
Yes	58	39 (67.2)	19 (32.8)	0.1260	0.722			
No	16	10 (62.5)	6 (37.5)					
GERD								
Yes	14	9 (64.3)	5 (35.7)	0.0287	0.865			
No	60	40 (66.7)	20 (33.3)					
SAHS								
Yes	23	13 (56.5)	10 (43.5)	1.4021	0.236			
No	51	36 (70.6)	15 (29.4)					
COPD								
Yes	19	10 (52.6)	9 (47.4)	2.1089	0.146			
No	55	39 (70.9)	16 (29.1)					
Age of diagnosis (years)	74	49 (66.2)	25 (33.8)		0.016		0.96	0.92-0.99
<18	10	8 (80)	2 (20)	0.9820	0.322			
>18	64	41(64.1)	23 (35.9)					
ICS (µg/day)	74	49 (66.2)	25 (33.8)		0.549			
OCS cycles per year								
Yes	55	35 (63.6)	20 (36.4)	0.6373	0.425			
No	19	14 (73.7)	5 (26.3)					
Baseline FEV1 (%)								
<80	42	24 (57.1)	18 (42.9)	3.7673	0.052	<80	2.88	1.01-9.11
>80	29	23 (79.3)	6 (20.7)					
Exacerbation in previous year								
Yes	47	28 (59.6)	19 (40.4)	2.5401	0.111			
No	27	21 (77.8)	6 (22.2)					
Basal blood eosinophils (cell/mcl)								
<300	36	19 (52.8)	17 (47.2)	3.266	0.071	<300	2.57	0.93-7.54
>300	31	23 (74.2)	8 (25.8)					
Baseline IgE (IU/Ml)	65	42 (64.6)	23 (35.4)		0.573			

Characteristics	N	Response		X^2	p-value	Ref. Cat	OR	CI 95%
		R N(%)	NR N(%)					
Years with Omalizumab								
<5	51	33 (64.7)	18 (35.3)	0.1673	0.683			
>5	23	16 (69.6)	7 (30.4)					
Change of BT								
Yes	36	24 (66.7)	12 (33.3)	0.0064	0.936			
No	38	25 (65.8)	13 (34.2)					

BMI, body mass index; GERD, gastroesophageal reflux disease; SAHS, sleep apnea-hypopnea syndrome; COPD, chronic obstructive pulmonary disease; ICS, inhaled corticosteroids; OCS, oral corticosteroids; FEV1, maximum expiratory volume in the first second of forced expiration; IgE, immunoglobulin E; BT, biological therapy.

Ref. Cat, Reference category; NR, Non-Responder; R, Responder; OR, Odds Ratio; CI 95%, Confidence interval; *p-value for Fisher's Exact Test.
