# Supplementary material for: Influence of Genetics on the Response to Omalizumab in Patients with Severe Uncontrolled Asthma with an Allergic Phenotype
Source: Int J Mol Sci. 2023 Apr 10;24(8):7029. doi: 10.3390/ijms24087029 (PMC10139019; doi:10.3390/ijms24087029)
Supplement: Supplementary file 1 [file ijms-24-07029-s001.zip › Table S13.pdf]

Table S13. Association of omalizumab genetic polymorphisms with corticosteroid reduction and/or absence.

Gene	SNPs	Genotype	N	Response R N (%)	Response NR N (%)	χ²	p-value	Ref Cat	OR	CI 95%
IL1RL1	rs1420101	CC	30	17 (56.7)	13 (43.3)	3.7827	0.151			
		CT	34	23 (67.6)	11 (32.4)					
		TT	10	9 (90)	1 (10)					
		C	64	40 (62.5)	24 (37.5)	2.9238	0.087			
		T	44	32 (72.7)	12 (27.3)	2.0568	0.152			
	rs17026974	AA	3	3 (100)	0 (0)		0.685			
		AG	28	18 (64.3)	10 (35.7)					
		GG	43	28 (65.1)	15 (34.9)					
		A	31	21 (67.7)	10 (32.3)	0.0555	0.814			
		G	71	46 (64.8)	25 (35.2)		0.547*			
	rs1921622	AA	9	8 (88.9)	1 (11.1)	2.5406	0.281			
		AG	48	31 (64.6)	17 (35.4)					
		GG	17	10 (58.8)	7 (41.8)					
		A	57	39 (68.4)	18 (31.6)	0.5392	0.463			
		G	65	41 (63.1)	24 (36.9)	2.3545	0.125			
GATA2	rs4857855	CC	55	34 (61.8)	21 (38.2)		0.241*			
		CT	17	14 (82.4)	3 (17.6)					
		TT	2	1 (50)	1 (50)					
		C	72	48 (66.7)	24 (33.3)		1*			
		T	19	15 (78.9)	4 (21.1)	1.8522	0.174			
FCER1A	rs2251746	CC	3	2 (66.7)	1 (33.3)		0.417*			
		CT	22	17 (77.3)	5 (22.7)					
		TT	49	30 (61.2)	19 (38.8)					
		C	25	19 (76)	6 (24)	1.6155	0.204			
		T	71	47 (66.2)	24 (33.8)		1*			
	rs2427837	AA	2	2 (100)	0 (0)		0.554*			
		AG	21	15 (71.4)	6 (28.6)					
		GG	51	32 (62.7)	19 (37.3)					
		A	23	17 (73.9)	6 (26.1)	0.8838	0.347			
		G	72	47 (65.3)	25 (34.7)		0.547*			
FCER1B	rs1441586	CC	17	12 (70.6)	5 (29.4)	0.2166	0.897			
		CT	42	27 (64.3)	15 (35.7)					
		TT	15	10 (66.7)	5 (33.3)					
		C	59	39 (66.1)	20 (33.9)	0.0017	0.967			
		T	57	37 (64.9)	20 (35.1)	0.1886	0.664			
	rs573790	CC	35	21 (60)	14 (40)	1.3343	0.5132			
		CT	30	21 (70)	9 (30)					
		TT	9	7 (77.8)	2 (22.2)					
		C	65	42 (64.6)	23 (35.4)	0.6122	0.434			
		T	39	28 (71.8)	11 (28.2)	1.1471	0.284			
	rs1054485	GG	24	16 (66.7)	8 (33.3)	0.2885	0.866			
		GT	39	(25 (64.1)	14 (35.9)					
		TT	11	8 (72.7)	3 (27.3)					
		G	63	41 (65.1)	22 (34.9)	0.2449	0.621			
		T	50	33 (66)	17 (34)	0.0032	0.955			
	rs569108	AA	67	43 (64.2)	24 (35.8)		0.411*			
		AG	7	6 (85.7)	1 (14.3)					
		GG	0	0 (0)	0 (0)					
		A	-	-	-					
		G	7	6 (85.7)	1 (14.3)		0.411*			
C3	rs2230199	CC	2	1 (50)	1 (50)		0.376*			
		CG	25	19 (76)	6 (24)					
		GG	47	29 (61.7)	18 (38.3)					
		C	27	20 (74.1)	7 (25.9)	1.1734	0.279			
		G	72	48 (66.7)	24 (33.3)	0.2416	0.623			
FCGR2A	rs1801274	AA	22	17 (77.3)	5 (22.7)	2.0345	0.362			
		AG	34	20 (58.8)	14 (41.2)					
		GG	18	12 (66.7)	6 (33.3)					
		A	56	37 (66.1)	19 (33.9)	0.0022	0.963			
		G	52	32 (61.5)	20 (38.5)	1.7109	0.191			

Gene	SNPs	Genotype	N	Response		χ²	p-value	Ref Cat	OR	CI 95%
				R<br>N (%)	NR<br>N (%)					
FCGR2B	rs3219018	CC	1	1 (100)	0 (0)		0.739*			
		CG	24	17 (70.8)	7 (29.2)					
		GG	49	31 (63.3)	18 (36.7)					
		C	25	18 (72)	7 (28)	0.5646	0.452			
		G	73	48 (65.8)	25 (34.2)		1*			
	rs1050501	CC	0	0 (0)	0 (0)	0.1754	0.675			
		CT	20	14 (70)	6 (30)					
		TT	54	35 (64.8)	19 (35.2)					
		C	20	14 (70)	6 (30)	0.1754	0.675			
		T	-	-	-					
FCGR3A	rs10127939	AA	68	46 (67.6)	22 (32.4)		0.557*			
		AC	5	2 (40)	3 (60)					
		CC	1	1 (100)	0 (0)					
		A	73	48 (65.8)	25 (34.2)		1*			
		C	6	3 (50)	3 (50)		0.40*			
	rs396991	AA	26	17 (65.4)	9 (34.6)	1.0162	0.602			
		CA	38	24 (63.2)	14 (36.8)					
		CC	10	8 (80)	2 (20)					
		A	64	41 (64.1)	23 (35.9)	0.9820	0.322			
		C	48	32 (66.7)	16 (33.3)	0.0124	0.911			

Ref. Cat., reference category; R, responder; NR, non-responder; OR, odds ratio; CI 95%, 95% confidence Interval 95%; *p-value for Fisher exact test.
