# Supplementary material for: Influence of Genetics on the Response to Omalizumab in Patients with Severe Uncontrolled Asthma with an Allergic Phenotype
Source: Int J Mol Sci. 2023 Apr 10;24(8):7029. doi: 10.3390/ijms24087029 (PMC10139019; doi:10.3390/ijms24087029)
Supplement: Supplementary file 1 [file ijms-24-07029-s001.zip › Table S14.pdf]

Table S14. Association of clinical characteristics of omalizumab-treated patients with improvement in lung function.

Characteristics	N	Response R N (%)	Response NR N (%)	X^2	p-value	Ref. Cat	OR	CI 95%
Sex								
Female	44	34 (77.3)	10 (22.7)	0.0446	0.833			
Male	24	18 (75)	6 (25)					
Age of initiation BT (years)	68	52 (76.5)	16 (23.5)		0.018		0.945	0.9-0.98
Years with asthma	68	52 (76.5)	16 (23.5)		0.929			
BMI (kg/m2)								
<25	15	14 (93.3)	1 (6.7)	3.1505	0.076	>25	5.68	1-107.39
>25	52	37 (71.2)	15 (28.8)					
Previous respiratory disease								
Yes	18	9 (50)	9 (50)	9.5331	0.002	Si	6.14	1.85-21.81
No	50	43 (86)	7 (14)					
Tobacco consumption								
Non smoker	50	37 (74)	13 (26)	1.4347	0.910*			
Current smoker	3	3 (100)	0 (0)					
Former smoker	15	12 (80)	3 (20)					
Polyps								
Yes	16	12 (75)	4 (25)	0.0251	0.874			
No	52	40 (76.9)	12 (23.1)					
Allergies								
Yes	52	41 (78.8)	11 (21.2)	0.6931	0.405			
No	16	11 (68.8)	5 (31.2)					
GERD								
Yes	13	4 (30.8)	9 (69.2)	1.623	0.203			
No	55	48 (87.3)	7 (12.7)					
SAHS								
Yes	21	14 (66.7)	7 (33.3)	0.0252	0.874			
No	47	38 (80.9)	9 (19.1)					
COPD								
Yes	16	12 (75)	4 (25)	18.657	<0.001	Si	15.43	3.98-71.29
No	52	40 (76.9)	12 (23.1)					
Age of diagnosis (years)	68	52 (76.5)	16 (23.5)		0.019		0.95	0.91-0.99
<18	9	9 (100)	0 (0)	3.1917	0.074			
>18	59	43 (72.9)	16 (27.1)					
ICS (µg/day)	68	52 (76.5)	16 (23.5)		0.132			
OCS cycles per year								
Yes	51	39 (76.5)	12 (23.5)	0	1			
No	17	13 (76.5)	4 (23.5)					
Baseline FEV1 (%)								
<80	41	29 (70.7)	12 (29.3)	2.637	0.104			
>80	25	22 (88)	3 (12)					
Exacerbation in previous year								
Yes	45	36 (80)	9 (20)	0.9211	0.337			
No	23	16 (69.6)	7 (30.4)					
Basal blood eosinophils (cell/mcl)								
<300	33	26 (78.8)	7 (21.2)	0.4424	0.506			
>300	28	20 (71.4)	8 (28.6)					
Baseline IgE (IU/Ml)	60	46 (76.7)	14 (23.3)		0.979			

Characteristics	N	Response		X^2	p-value	Ref. Cat	OR	CI 95%
		R N(%)	NR N(%)					
Years with Omalizumab								
<5	45	4 (75.6)	11 (24.4)	0.0619	0.804			
>5	23	18 (78.3)	5 (21.7)					
Previous BT								
Yes	2	0 (0)	2 (100)		0.052*			
No	66	52 (78.8)	14 (21.2)					

BMI, body mass index; GERD, gastroesophageal reflux disease; SAHS, sleep apnea-hypopnea syndrome; COPD, chronic obstructive pulmonary disease; ICS, inhaled corticosteroids; OCS, oral corticosteroids; FEV1, maximum expiratory volume in the first second of forced expiration; IgE, immunoglobulin E; BT, biological therapy.

Ref. Cat, Reference category; NR, Non-Responder; R, Responder; OR, Odds Ratio; CI 95%, Confidence interval; *p-value for Fisher's Exact Test.
