# Supplementary material for: Influence of Genetics on the Response to Omalizumab in Patients with Severe Uncontrolled Asthma with an Allergic Phenotype
Source: Int J Mol Sci. 2023 Apr 10;24(8):7029. doi: 10.3390/ijms24087029 (PMC10139019; doi:10.3390/ijms24087029)
Supplement: Supplementary file 1 [file ijms-24-07029-s001.zip › Table S15.pdf]

Table S15. Association of omalizumab genetic polymorphisms with improved lung function (FEV1).

Gene	SNPs	Genotype	N	Response R N (%)	Response NR N (%)	χ²	p-value	Ref Cat	OR	CI 95%
IL1RL1	rs1420101	CC	28	22 (78.6)	6 (21.4)	3.064	0.216			
		CT	33	23 (69.7)	10 (30.3)					
		TT	7	7 (100)	0 (0)					
		C	61	45 (73.8)	16 (26.2)	2.401	0.121			
		T	28	22 (78.6)	6 (21.4)	0.1168	0.733			
	rs17026974	AA	3	3 (100)	0 (0)		0.089*			
		AG	27	17 (63)	10 (37)					
		GG	38	32 (84.2)	6 (15.8)					
		A	30	20 (66.7)	10 (33.3)	2.8677	0.148*			
		G	65	49 (75.4)	16 (24.6)	0.9657	1*			
	rs1921622	AA	9	9 (100)	0 (0)		0.195*			
		AG	43	32 (74.4)	11 (25.6)					
		GG	16	11 (68.8)	5 (31.2)					
		A	52	41 (78.8)	11 (21.2)	0.6931	0.405			
		G	59	43 (72.9)	16 (27.1)	3.1917	0.074			
GATA2	rs4857855	CC	52	37 (71.2)	15 (28.8)		0.204*			
		CT	14	13 (92.9)	1 (7.1)					
		TT	2	2 (100)	0 (0)					
		C	66	50 (75.8)	16 (24.2)		1*			
		T	16	15 (93.8)	1 (6.2)	3.472	0.062	CC	6.08	1.07-114.8
	rs2251746	CC	3	1 (33.3)	2 (66.7)		0.228*			
		CT	18	14 (77.8)	4 (22.2)					
		TT	47	37 (78.7)	10 (21.3)					
		C	21	15 (71.4)	6 (28.6)	0.4293	0.512			
		T	65	51 (78.5)	14 (21.5)		0.136*			
FCER1A	rs2427837	AA	2	1(50)	1 (50)		0.362*			
		AG	18	13 (72.2)	5 (27.8)					
		GG	48	38 (79.2)	10 (20.8)					
		A	20	14 (70)	6 (30)	0.6593	0.417			
		G	66	51 (77.3)	15 (22.7)		0.418*			
FCER1B	rs1441586	CC	14	11 (78.6)	3 (21.4)		1*			
		CT	39	30 (76.9)	9 (33.1)					
		TT	15	11 (73.3)	4 (26.7)					
		C	53	41 (77.4)	12 (22.6)	0.1053	0.746			
		T	54	41 (75.9)	13 (24.1)	0.0432	0.835			
	rs573790	CC	32	26 (81.2)	6 (18.8)	0.9732	0.615			
		CT	27	19 (70.4)	8 (29.6)					
		TT	9	7 (77.8)	2 (22.2)					
		C	59	45 (76.3)	14 (23.7)	0.0099	0.921			
		T	32	26 (81.2)	4 (18.8)	0.7674	0.381			
	rs1054485	GG	22	18 (81.8)	4 (18.2)	1.3654	0.505			
		GT	35	27 (77.1)	8 (22.9)					
		TT	11	7 (63.6)	4 (36.4)					
		G	57	45 (78.9)	12 (21.1)	1.2013	0.273			
		T	46	34 (73.9)	12 (26.1)	0.5169	0.472			
	rs569108	AA	63	48 (76.2)	15 (23.8)		1*			
		AG	5	4 (80)	1 (20)					
		GG	0	0 (0)	0 (0)					
		A	-	-	-					
		G	5	4 (80)	1 (20)		1*			
C3	rs2230199	CC	2	2 (100)	0 (0)		0.312*			
		CG	22	19 (86.4)	3 (13.6)					
		GG	44	31 (70.5)	13 (29.5)					
		C	24	21 (87.5)	3 (12.5)	2.5076	0.113			
		G	66	50 (75.8)	19 (24.2)		1*			

Gene	SNPs	Genotype	N	Response		χ²	p-value	Ref Cat	OR	CI 95%
				R N (%)	NR N (%)					
FCGR2A	rs1801274	AA	21	12 (57.1)	9 (42.9)		0.052*	AA		
		AG	32	28 (87.5)	4 (12.5)				5.25	1.42-22.64
		GG	15	12 (80)	3 (20)				3	0.7-16.13
		A	53	40 (75.5)	13 (24.5)	0.1332	0.715			
		G	47	40 (85.1)	7 (14.9)	6.3079	0.012	AA	4.29	1.33-14.49
FCGR2B	rs3219018	CC	1	1 (100)	0 (0)		0.104*			
		CG	21	19 (90.5)	2 (9.5)					
		GG	46	32 (69.4)	14 (30.4)					
		C	22	20 (90.9)	2 (9.1)	3.768	0.052	GG	4.37	1.07-29.76
		G	1	1 (100)	0 (0)		1*			
	rs1050501	CC	0	0 (0)	0 (0)	0.6593	0.417			
		CT	20	14 (70)	6 (30)					
		TT	48	38 (79.2)	10 (20.8)					
		C	20	14 (70)	6 (30)	0.6593	0.417			
		T	-	-	-					
FCGR3A	rs10127939	AA	62	47 (75.8)	15 (24.2)		1*			
		AC	5	4 (80)	1 (20)					
		CC	1	1 (100)	0 (0)					
		A	67	51 (76.1)	16 (23.9)		1*			
		C	6	5 (83.3)	1 (16.7)		1*			
	rs396991	AA	25	16 (64)	9 (36)	5.2569	0.072	AA		
		CA	34	30 (88.2)	4 (11.8)				4.22	1.18-17.63
		CC	9	6 (66.7)	3 (33.3)				1.13	0.23-6.35
		A	59	46 (78)	13 (22)	0.5541	0.457			
		C	43	36 (83.7)	7 (16.3)	3.417	0.065			

Ref. Cat., reference category; R, responder; NR, non-responder; OR, odds ratio; CI 95%, 95% confidence Interval 95%; *p-value for Fisher exact test.
