# Supplementary material for: Influence of Genetics on the Response to Omalizumab in Patients with Severe Uncontrolled Asthma with an Allergic Phenotype
Source: Int J Mol Sci. 2023 Apr 10;24(8):7029. doi: 10.3390/ijms24087029 (PMC10139019; doi:10.3390/ijms24087029)
Supplement: Supplementary file 1 [file ijms-24-07029-s001.zip › Table S16.pdf]

Table S16. Association of clinical characteristics of omalizumab-treated patients with response to at least one parameter.

Characteristics	N	Response		X^2	p-value	Ref. Cat	OR	CI 95%
		R N (%)	NR N (%)					
Sex								
Female	48	46 (95.8)	2 (4.2)	0.0045	1*			
Male	26	25 (96.2)	1 (3.8)					
Age of initiation BT (years)	74	71 (95.9)	3 (4.1)		0.061		0.87	0.73-0.98
Years with asthma	74	71 (95.9)	3 (4.1)		0.355			
BMI (kg/m2)								
<25	17	17 (100)	0 (0)	0.9676	1*			
>25	55	52 (94.5)	3 (5.5)					
Previous respiratory disease								
Yes	19	17 (8.5)	2 (10.5)	2.7531	0.16*			
No	55	54 (98.2)	1 (1.8)					
Tobacco consumption								
Non smoker	55	53 (96.4)	2 (3.6)	0.4569	0.595*			
Current smoker	3	3 (100)	0 (0)					
Former smoker	16	15 (93.8)	1 (6.2)					
Polyps								
Yes	18	18 (100)	0 (0)	1.005	1*			
No	56	53 (94.6)	3 (5.4)					
Allergies								
Yes	58	56 (96.6)	2 (3.4)	0.2531	0.524*			
No	16	15 (93.8)	1 (6.2)					
GERD								
Yes	14	11 (78.6)	3 (21.4)	13.4	0.006*	Si	$6.3e^{8}$	$1.18e^{-212}$-NA
No	60	60 (100)	0 (0)					
SAHS								
Yes	23	22 (95.7)	1 (4.3)	0.0074	1*			
No	51	49 (96.1)	2 (3.9)					
COPD								
Yes	19	19 (100)	0 (0)	1.0802	0.565*			
No	55	52 (94.5)	3 (5.5)					
Age of diagnosis (years)	74	71 (95.9)	3 (4.1)		0.036		0.86	0.72-0.97
<18	10	10 (100)	0 (0)	0.4886	1*			
>18	64	61 (95.3)	3 (4.7)					
ICS (µg/day)	74	71 (95.9)	3 (4.1)		0.766			
OCS cycles per year								
Yes	55	54 (98.2)	1 (1.8)	2.7531	0.16*			
No	19	17 (89.5)	2 (10.5)					
Baseline FEV1 (%)								
<80	42	39 (92.9)	3 (7.1)	2.1628	0.265*			
>80	29	29 (100)	0 (0)					
Exacerbation in previous year								
Yes	47	46 (97.9)	1 (2.1)	1.229	0.55*			
No	27	25 (92.6)	2 (7.4)					
Basal blood eosinophils (cell/mcl)								
<300	36	34 (94.4)	2 (5.6)	0.2114	1*			
>300	31	30 (96.8)	1 (3.2)					
Baseline IgE (IU/Ml)	65	62 (95.4)	3 (4.6)		0.598			

Characteristics	N	Response R N (%)	Response NR N (%)	X^2	p-value	Ref. Cat	OR	CI 95%
Years with Omalizumab								
<5	51	48 (94.1)	3 (5.9)	1.4101	0.548*			
>5	23	23 (100)	0 (0)					
Change of BT								
Yes	36	33 (91.7)	3 (8.3)	3.3005	0.11*			
No	38	38 (100)	0 (0)					

BMI, body mass index; GERD, gastroesophageal reflux disease; SAHS, sleep apnea-hypopnea syndrome; COPD, chronic obstructive pulmonary disease; ICS, inhaled corticosteroids; OCS, oral corticosteroids; FEV1, maximum expiratory volume in the first second of forced expiration; IgE, immunoglobulin E; BT, biological therapy.

Ref. Cat, Reference category; NR, Non-Responder; R, Responder; OR, Odds Ratio; CI 95%, Confidence interval; *p-value for Fisher's Exact Test.
