# Supplementary material for: Influence of Genetics on the Response to Omalizumab in Patients with Severe Uncontrolled Asthma with an Allergic Phenotype
Source: Int J Mol Sci. 2023 Apr 10;24(8):7029. doi: 10.3390/ijms24087029 (PMC10139019; doi:10.3390/ijms24087029)
Supplement: Supplementary file 1 [file ijms-24-07029-s001.zip › Table S17.pdf]

Table S17. Association of omalizumab genetic polymorphisms with responders to at least one criterion.

Gene	SNPs	Genotype	N	Response R N (%)	Response NR N (%)	χ²	p-value	Ref Cat	OR	CI 95%
IL1RL1	rs1420101	CC	30	29 (96.7)	1 (3.3)		1*			
		CT	34	32 (94.1)	2 (5.9)					
		TT	10	10 (100)	0 (0)					
		C	64	61 (95.3)	3 (4.7)		1*			
		T	44	42 (95.5)	2 (4.5)		1*			
	rs17026974	AA	3	3 (100)	0 (0)		0.61*			
		AG	28	26 (92.9)	2 (7.1)					
		GG	43	42 (97.7)	1 (2.3)					
		A	31	29 (93.5)	2 (6.5)		0.568*			
		G	71	68 (95.8)	3 (4.2)		1*			
	rs1921622	AA	9	9 (100)	0 (0)		0.704*			
		AG	48	45 (93.8)	3 (6.3)					
		GG	17	17 (100)	0 (0)					
		A	57	54 (94.7)	3 (5.3)		1*			
		G	65	62 (95.4)	3 (4.6)		1*			
GATA2	rs4857855	CC	55	52 (94.5)	3 (5.5)		1*			
		CT	17	17 (100)	0 (0)					
		TT	2	2 (100)	0 (0)					
		C	72	69 (95.8)	3 (4.2)		1*			
		T	19	19 (100)	0 (0)		0.565*			
FCER1A	rs2251746	CC	3	2 (66.7)	1 833.3)		0.088*	CC		
		CT	22	21 (95.5)	1 (4.5)				10.5	0.33-356.19
		TT	49	48 (98)	1 (2)				24	0.77-804.57
		C	25	23 (92)	2 (8)		0.262*			
		T	71	69 (97.2)	2 (2.8)		0.008*	CC	17.25	0.65-291.88
	rs2427837	AA	2	2 (100)	0 (0)	2.2743	0.51			
		AG	21	19 (90.5)	2 (9.5)					
		GG	51	50 (98)	1 (2)					
		A	23	21 (91.3)	2 (9.7)		0.226*			
		G	72	69 (95.8)	3 (4.2)		1*			
FCER1B	rs1441586	CC	17	17 (100)	0 (0)		0.774*			
		CT	42	40 (95.2)	2 (1.8)					
		TT	15	14 (93.3)	1 (6.7)					
		C	59	57 (96.6)	2 (3.4)		0.499*			
		T	57	54 (954.7)	3 (5.3)		1*			
	rs573790	CC	35	34 (97.1)	1 (2.9)		0.725*			
		CT	30	28 (93.3)	2 (6.7)					
		TT	9	9 (100)	0 (0)					
		C	65	62 (95.4)	3 (4.6)		1*			
		T	39	37 (94.9)	2 (5.1)		1*			
	rs1054485	GG	24	23 (95.8)	1 (4.2)		1*			
		GT	39	37 (94.9)	2 (5.1)					
		TT	11	11 (100)	0 (0)					
		G	63	60 (95.2)	3 (4.8)		1*			
		T	50	48 (96)	2 (4)		1*			
	rs569108	AA	67	64 (95.5)	3 (4.5)		1*			
		AG	7	7 (100)	0 (0)					
		GG	0	0 (0)	0 (0)					
		A	-	-	-					
		G	7	7 (100)	0 (0)		1*			
	rs2230199	CC	2	2 (100)	0 (0)		0.583*			
		CG	25	25 (100)	0 (0)					
		GG	47	44 (93.6)	3 (6.4)					
		C	27	27 (100)	0 (0)		0.295*			
		G	72	69 (95.8)	3 (4.2)		1*			

Gene	SNPs	Genotype	N	Response		χ²	p-value	Ref Cat	OR	CI 95%
				R N (%)	NR N (%)					
FCGR2A	rs1801274	AA	22	20 (90.9)	2 (9.1)		0.152*			
		AG	34	34 (100)	0 (0)					
		GG	18	17 (94.4)	1 (5.6)					
		A	56	54 (96.4)	2 (3.6)		1*			
		G	52	51 (98.1)	1 (1.9)		0.209*			
FCGR2B	rs3219018	CC	1	1 (100)	0 (0)		0.565*			
		CG	24	24 (100)	0 (0)					
		GG	49	46 (93.9)	3 (6.1)					
		C	25	25 (100)	0 (0)		0.547*			
		G	73	70 (95.9)	3 (4.1)		1*			
	rs1050501	CC	0	0 (0)	0 (0)		1*			
		CT	20	19 (95)	1 (5)					
		TT	54	52 (96.3)	2 (3.7)					
		C	20	19 (95)	1 (5)		1*			
		T	-	-	-					
	rs10127939	AA	68	65 (95.6)	3 (4.4)		1*			
		AC	5	5(100)	0 (0)					
		CC	1	1 (100)	0 (0)					
		A	73	70 (95.9)	3 (4.1)		1*			
		C	6	6 (100)	0 (0)		1*			
	rs396991	AA	26	25 (96.2)	1 (3.8)		1*			
		CA	38	36 (94.7)	2 (5.3)					
		CC	10	10 (100)	0 (0)					
		A	64	61 (95.3)	3 (4.7)		1*			
		C	48	46 (95.8)	2 (4.2)		1*			

Ref. Cat., reference category; R, responder; NR, non-responder; OR, odds ratio; CI 95%, 95% confidence Interval 95%; *p-value for Fisher exact test.
