# Supplementary material for: Influence of Genetics on the Response to Omalizumab in Patients with Severe Uncontrolled Asthma with an Allergic Phenotype
Source: Int J Mol Sci. 2023 Apr 10;24(8):7029. doi: 10.3390/ijms24087029 (PMC10139019; doi:10.3390/ijms24087029)
Supplement: Supplementary file 1 [file ijms-24-07029-s001.zip › Table S18.pdf]

Table S18. Association of clinical characteristics of patients treated with omalizumab with response on at least 2 criteria.

Characteristics	N	Response		X^2	p-value	Ref. Cat	OR	CI 95%
		R<br>N (%)	NR<br>N (%)					
Sex								
Female	48	42 (87.5)	6 (12.5)	0.6037	0.437			
Male	26	21 (80.8)	5 819.2)					
Age of initiation BT (years)	74	63 (85.1)	11 (14.9)		0.026		0.94	0.88-0.99
Years with asthma	74	63 (85.1)	11 (14.9)		0.287			
BMI (kg/m2)								
<25	17	17 (100)	0 (0)	4.0131	0.045	2.8e[6]	>25	9.88e-46-NA
>25	55	44 (80)	11 (20)					
Previous respiratory disease								
Yes	19	14 (73.7)	5 (26.3)	2.6487	0.104			
No	55	49 (89.1)	6 (10.9)					
Tobacco consumption								
Non-smoker	55	47 (85.5)	8 (14.5)		0.603*			
Current smoker	3	2 (66.7)	1 (33.6)					
Former smoker	16	14 (87.5)	2 (12.5)					
Polyps								
Yes	18	14 (77.8)	4 (22.2)	1.0174	0.313			
No	56	49 (87.5)	7 (12.5)					
Allergies								
Yes	58	48 (82.8)	10 (17.2)	1.1972	0.274			
No	16	15 (93.8)	1 (6.2)					
GERD								
Yes	14	10 (71.4)	4 (28.6)	2.5633	0.109			
No	60	53 (88.3)	7 (11.7)					
SAHS								
Yes	23	18 (78.3)	5 (21.7)	1.2462	0.264			
No	51	45 (88.2)	6 (11.8)					
COPD								
Yes	19	15 (78.9)	4 (21.1)	0.7734	0.379			
No	55	48 (87.3)	7 (12.7)					
Age of diagnosis (years)	74	63 (85.1)	11 (14.9)		0.018		0.93	0.88-0.99
<18	10	10 (100)	0 (0)	2.0188	0.155			
>18	64	53 (82.8)	11 (17.2)					
ICS (µg/day)	74	63 (85.1)	11 (14.9)		0.261			
OCS cycles per year								
Yes	55	46 (83.6)	9 (16.4)	0.3802	0.538			
No	19	17 (89.5)	2 (10.5)					
Baseline FEV1 (%)								
<80	42	34 (81)	8 (19)	2.0932	0.148			
>80	29	27 (93.1)	2 (6.9)					
Exacerbation in previous year								
Yes	47	38 (80.9)	9(19.1)	1.8681	0.172			
No	27	25 (92.6)	2 (7.4)					
Basal blood eosinophils (cell/mcl)								
<300	36	28 (77.8)	8 (22.2)	1.9102	0.167			
>300	31	28 (90.3)	3 (9.7)					
Baseline IgE (IU/Ml)	64	54 (84.4)	11 (15.6)		0.512			

Characteristics	N	Response		X^2	p-value	Ref. Cat	OR	CI 95%
		R<br>N (%)	NR<br>N (%)					
Years with Omalizumab								
<5	51	42 (82.4)	9 (17.6)	1.0036	0.316			
>5	23	21 (91.3)	2 (8.7)					
Change of BT								
Yes	36	29 (80.6)	7 (19.4)	1.1618	0.281			
No	38	34 (89.5)	4 (10.5)					

BMI, body mass index; GERD, gastroesophageal reflux disease; SAHS, sleep apnea-hypopnea syndrome; COPD, chronic obstructive pulmonary disease; ICS, inhaled corticosteroids; OCS, oral corticosteroids; FEV1, maximum expiratory volume in the first second of forced expiration; IgE, immunoglobulin E; BT, biological therapy.

Ref. Cat, Reference category; NR, Non-Responder; R, Responder; OR, Odds Ratio; CI 95%, Confidence interval; *p-value for Fisher's Exact Test.
