# Supplementary material for: Influence of Genetics on the Response to Omalizumab in Patients with Severe Uncontrolled Asthma with an Allergic Phenotype
Source: Int J Mol Sci. 2023 Apr 10;24(8):7029. doi: 10.3390/ijms24087029 (PMC10139019; doi:10.3390/ijms24087029)
Supplement: Supplementary file 1 [file ijms-24-07029-s001.zip › Table S19.pdf]

Table S19. Association of omalizumab genetic polymorphisms with 2-criteria response.

Gene	SNPs	Genotype	N	Response		χ²	p-value	Ref Cat	OR	CI 95%
				R N (%)	NR N (%)					
IL1RL1	rs1420101	CC	30	24 (80)	6 (20)		0.441*			
		CT	34	29 (85.3)	5 (14.7)					
		TT	10	10 (100)	0 (0)					
		C	64	53 (82.8)	11 (17.2)	2.0188	0.155			
		T	44	39 (88.6)	5 (11.4)	1.0513	0.305			
	rs17026974	AA	3	3 (100)	0 (0)	0.59991				
		AG	28	24 (85.7)	4 (14.3)					
		GG	43	36 (83.7)	7 (16.3)					
		A	31	27 (87.1)	4 (12.9)	0.1622	0.687			
		G	71	60 (84.5)	11 (155)		1*			
	rs1921622	AA	9	9 (100)	0 (0)		0.561*			
		AG	48	40 (83.3)	8 (16.7)					
		GG	17	14 (82.4)	3 (17.6)					
		A	57	49 (82.4)	8 (17.6)	0.135	0.713			
		G	65	54 (83.1)	11 (16.9)	1.789	0.181			
GATA2	rs4857855	CC	55	44 (80)	11 (20)		0.088*			
		CT	17	17 (100)	0 (0)					
		TT	2	2 (100)	0 (0)					
		C	72	61 (84.7)	11 (15.3)		1*			
		T	19	19 (100)	0 (0)	4.4635	0.035	CC	$7.8e^{7}$	1.18e-74-NA
FCER1A	rs2251746	CC	3	2 (66.7)	1 (33.3)		0.57*			
		CT	22	19 (86.4)	3 (13.6)					
		TT	49	42 (85.7)	7 (14.3)					
		C	25	21 (84)	4 (16)	0.0384	0.845			
		T	71	61 (85.9)	10 (14.1)	0.8427	0.387			
	rs2427837	AA	2	2 (100)	0 (0)		0.797*			
		AG	21	17 (81)	4 (19)					
		GG	51	44 (86.3)	7 (13.7)					
		A	23	19 (82.6)	4 (17.4)	0.1683	0.682			
		G	72	61 (84.7)	11 (15.3)		1*			
FCER1B	rs1441586	CC	17	15 (88.2)	2 (11.8)		1*			
		CT	42	35 (83.3)	7 (16.7)					
		TT	15	13 (86.7)	2 (13.3)					
		C	59	50 (84.7)	9 (15.3)	0.0349	0.852			
		T	57	48 (84.2)	9 (15.8)	0.1676	0.682			
	rs573790	CC	35	31 (88.6)	4 (11.4)		0.728*			
		CT	30	24 (80)	6 (20)					
		TT	9	8 (88.9)	1 (11.1)					
		C	65	55 (84.6)	10 (15.4)	0.1141	0.736			
		T	39	32 (82.1)	7 (17.9)	0.6197	0.431			
	rs1054485	GG	24	22 (91.7)	2 (8.3)		0.447*			
		GT	39	31 (79.5)	8 (20.5)					
		TT	11	10 (90.9)	1 (9.1)					
		G	63	53 (94.1)	10 (15.9)	0.3404	0.56			
		T	50	41 (82)	9 (18)	1.1974	0.2774			
	rs569108	AA	67	56 (83.6)	11 (16.4)	1.3499	0.245			
		AG	7	7 (100)	0 (0)					
		GG	0	0 (0)	0 (0)					
		A	-	-	-					
		G	7	7 (100)	0 (0)	1.3499	0.245			
C3	rs2230199	CC	2	2 (100)	0 (0)		0.5*			
		CG	25	23 (92)	2 (8)					
		GG	47	38 (80.9)	9 (19.1)					
		C	27	25 (92.6)	2 (7.4)	1.8681	0.172			
		G	72	61 (84.7)	11 (15.3)		1*			
FCGR2A	rs1801274	AA	22	18 (81.8)	4 (18.2)		0.841*			
		AG	34	29 (85.3)	5 (14.7)					
		GG	18	16 (88.9)	2 (11.1)					
		A	56	47 (83.9)	9 (16.1)	0.2648	0.607			
		G	52	45 (86.5)	7 (13.5)	0.2722	0.602			

Gene	SNPs	Genotype	N	Response		χ^2	p-value	Ref Cat	OR	CI 95%
				R N (%)	NR N (%)					
FCGR2B	rs3219018	CC	1	1 (100)	0 (0)		0.421*			
		CG	24	22 (91.7)	2 (8.3)					
		GG	49	40 (81.6)	9 (18.4)					
		C	25	23 (92)	2 (8)	1.4059	0.236			
		G	73	62 (84.9)	11 (15.1)		1*			
	rs1050501	CC	0	0 (0)	0 (0)	0.0004	0.984			
		CT	20	17 (85)	3 (25)					
		TT	54	46 (85.2)	8 (14.8)					
		C	20	17 (85)	3 (25)					
		T	-	-	-					
FCGR3A	rs10127939	AA	68	58 (85.3)	10 (14.7)	0.2802	0.633*			
		AC	5	4 (80)	1 (20)					
		CC	1	1 (100)	0 (0)					
		A	73	62 (94.9)	11 (15.1)		1*			
		C	6	5 (83.3)	1 (16.7)	0.0168	0.897			
	rs396991	AA	26	21 (80.8)	5 (19.2)		0.444*			
		CA	38	32 (84.2)	6 (15.8)					
		CC	10	10 (100)	0 (0)					
		A	64	53 (82.8)	11 (17.2)	2.0188	0.155			
		C	48	42 (87.5)	6 (12.5)	0.6037	0.437			

Ref. Cat., reference category; R, responder; NR, non-responder; OR, odds ratio; CI 95%, 95% confidence Interval 95%; *p-value for Fisher exact test.
