# Supplementary material for: Influence of Genetics on the Response to Omalizumab in Patients with Severe Uncontrolled Asthma with an Allergic Phenotype
Source: Int J Mol Sci. 2023 Apr 10;24(8):7029. doi: 10.3390/ijms24087029 (PMC10139019; doi:10.3390/ijms24087029)
Supplement: Supplementary file 1 [file ijms-24-07029-s001.zip › Table S2.pdf]

Table S2. Linkage disequilibrium of the studied SNPs.

CHR	BP	SNP	CHR	BP	SNP	R2	D'
1	159288755	rs2427837	1	159302270	rs2251746	0.877021	1
1	161548543	rs10127939	1	161662856	rs3219018	0.271969	1
2	102335900	rs17026974	2	102341256	rs1420101	0.310769	0,944018
2	102335900	rs17026974	2	102349607	rs1921622	0.262214	1
2	102341256	rs1420101	2	102349607	rs1921622	0.452584	0,845153
11	60087912	rs573790	11	60088555	rs1441586	0.487312	0,95104

BP, physical position (base pairs); CHR, chromosome; SNP, single nucleotide polymorphism.
