# Supplementary material for: Influence of Genetics on the Response to Omalizumab in Patients with Severe Uncontrolled Asthma with an Allergic Phenotype
Source: Int J Mol Sci. 2023 Apr 10;24(8):7029. doi: 10.3390/ijms24087029 (PMC10139019; doi:10.3390/ijms24087029)
Supplement: Supplementary file 1 [file ijms-24-07029-s001.zip › Table S20.pdf]

Table S20. Association of clinical characteristics of omalizumab-treated patients with response to the 3 criteria.

Characteristics	N	Response R N (%)	Response NR N (%)	X^2	p-value	Ref. Cat	OR	CI 95%
Sex								
Female	44	22 (50)	22 (50)	0.9782	0.323			
Male	24	9 (37.5)	15 (62.5)					
Age of initiation BT (years)	68	37 (54.4)	31 (45.6)		0.01		0.96	0.92-0.99
Years with asthma	68	37 (54.4)	31 (45.6)		0.743			
BMI (kg/m2)								
<25	15	13 (86.7)	2 (13.3	13.716	<0.001	>25	13.38	3.23-92.3
>25	52	17 (32.7)	35 (67.3)					
Previous respiratory disease								
Yes	18	4 (22.2)	14 (77.8)	5.3881	0.020	Si	4.11	1.27-16.1
No	50	27 (54)	23 (46)					
Tobacco consumption								
Non-smoker	50	20 (40)	30 (60)	3.9777	0.246*			
Current smoker	3	1 (33.3)	2 (66.7)					
Former smoker	15	10 (66.7)	5 (33.3)					
Polyps								
Yes	16	3 (18.8)	13 (81.2)	6.0756	0.014	Si	5.05	1.43-23.94
No	52	28 (53.8)	24 (46.2)					
Allergies								
Yes	52	25 (48.1)	27 (51.9)	0.5518	0.458			
No	16	6 (37.5)	10 (62.5)					
GERD								
Yes	13	3 (23.1)	10 (76.9)	3.2836	0.069	Si	3.46	0.94-16.67
No	55	28 (50.9)	27 (49.1)					
SAHS								
Yes	21	4 (19)	17 (81)	8.6279	0.003	Si	5.74	1.80-22.36
No	47	27 (57.4)	20 (42.6)					
COPD								
Yes	16	3 (18.8)	13 (81.2)	6.0756	0.014	Si	5.05	1.43-23.98
No	52	28 (53.8)	24 (46.2)					
Age of diagnosis (years)	68	37 (54.4)	31 (45.6)		0.006		0.95	0.92-0.98
<18	9	7 (77.8)	2 (22.2)	4.3329	0.069*	>18	5.10	1.12-36.29
>18	59	24 (40.7)	32 (59.3)					
ICS (µg/day)	68	37 (54.4)	31 (45.6)		0.436			
OCS cycles per year								
Yes	51	22 (43.1)	29 (56.9)	0.4940	0.482			
No	17	9 (52.9)	8 (47.1)					
Baseline FEV1 (%)								
<80	41	14 (34.1)	27 (65.9)	5.5826	0.018	>80	3.43	1.24-10.06
>80	25	16 (64)	9 (36)					
Exacerbation in previous year								
Yes	45	21 (46.7)	24 (53.3)	0.0624	0.803			
No	23	10 (43.5)	13 (56.5)					
Basal blood eosinophils (cell/mcl)								
<300	33	13 (39.4)	20 (60.6)	0.0751	0.784			
>300	28	12 (42.9)	16 (57.1)					
Baseline IgE (IU/Ml)	60	32 (53.3)	28 (46.7)		0.531			

Characteristics	N	Response		X^2	p-value	Ref. Cat	OR	CI 95%
		R<br>N (%)	NR<br>N (%)					
Years with Omalizumab								
<5	45	19 (42.2)	26 (57.8)	0.6077	0.436			
>5	23	12 (52.2)	11 (47.8)					
Change of BT								
Yes	33	12 (36.4)	21 (63.6)	2.1994	0.138			
No	35	19 (54.3)	16 (45.7)					

BMI, body mass index; GERD, gastroesophageal reflux disease; SAHS, sleep apnea-hypopnea syndrome; COPD, chronic obstructive pulmonary disease; ICS, inhaled corticosteroids; OCS, oral corticosteroids; FEV1, maximum expiratory volume in the first second of forced expiration; IgE, immunoglobulin E; BT, biological therapy.

Ref. Cat, Reference category; NR, Non-Responder; R, Responder; OR, Odds Ratio; CI 95%, Confidence interval; *p-value for Fisher's Exact Test.
