# Supplementary material for: Influence of Genetics on the Response to Omalizumab in Patients with Severe Uncontrolled Asthma with an Allergic Phenotype
Source: Int J Mol Sci. 2023 Apr 10;24(8):7029. doi: 10.3390/ijms24087029 (PMC10139019; doi:10.3390/ijms24087029)
Supplement: Supplementary file 1 [file ijms-24-07029-s001.zip › Table S21.pdf]

Table S21. Association of omalizumab genetic polymorphisms with response to the 3 criteria.

Gene	SNPs	Genotype	N	Response R N (%)	Response NR N (%)	χ²	p-value	Ref Cat	OR	CI 95%
IL1RL1	rs1420101	CC	28	12 (42.9)	16 (57.1)		0.829*			
		CT	33	15 (45.5)	18 (54.5)					
		TT	7	4 (57.1)	3 (42.9)					
		C	61	27 (44.3)	34 (55.7)		0.694*			
		T	40	19 (47.5)	21 (52.5)	0.1431	0.705			
	rs17026974	AA	3	1 (33.3)	2 (66.7)		0.444*			
		AG	27	10 (37)	17 (63)					
		GG	38	20 (52.6)	18 (47.4)					
		A	30	11 (36.7)	19 (63.3)	1.7226	0.189			
		G	65	30 (46.2)	35 (53.8)		1*			
	rs1921622	AA	9	6 (66.7)	3 (33.3)		0.379*			
		AG	46	19 (44.2)	24 (5.8)					
		GG	16	6 (37.5)	10 (62.5)					
		A	52	25 (48.1)	27 (51.9)	0.5518	0.458			
		G	9	6 (66.7)	3 (33.3)	1.8579	0.282			
GATA2	rs4857855	CC	52	21 (40.4)	31 (59.6)		0.210*			
		CT	14	9 (64.3)	5 (35.7)					
		TT	2	1 (50)	1 (50)					
		C	66	30 (45.5)	36 (54.5)	0.0162	0.899			
		T	16	10 (62.5)	6 (37.5)	2.4124	0.12			
FCER1A	rs2251746	CC	3	1 (33.3)	2(66.7)	0.3384	0.911			
		CT	18	9 (50)	9 (50)					
		TT	47	21 (44.7)	26 (55.3)					
		C	21	2110 (47.6)	11 (52.4)	0.0505	0.822			
		T	65	30 (46.2)	35 (53.8)		1*			
	rs2427837	AA	2	1 (50)	1 (50)		1*			
		AG	18	8 (44.4)	10 (55.6)					
		GG	48	22 (45.8)	26 (54.2)					
		A	20	9 (45)	11 (55)	0.004	0.95			
		G	66	30 (45.5)	36 (54.5)	0.0161	0.899			
FCER1B	rs1441586	CC	14	7 (50)	7 (50)	0.3072	0.859			
		CT	39	18 (46.2)	21 (53.8)					
		TT	15	6 (40)	9 (60)					
		C	53	25 (47.2)	28 (52.8)	0.2423	0.623			
		T	54	24 (44.4)	30 (55.6)	0.1383	0.71			
	rs573790	CC	32	15 (46.9)	17 (53.1)		1*			
		CT	27	12 (44.4)	15 (55.6)					
		TT	9	4 (44.4)	5 (55.6)					
		C	59	27 (45.8)	32 (54.2)		1*			
		T	36	16 (44.4)	20 (55.6)	0.403	0.841			
	rs1054485	GG	22	11 (50)	11 (50)	1.7858	0.41			
		GT	35	18 (51.4)	17 (48.6)					
		TT	11	3 (27.3)	8 (72.7)					
		G	57	28 (49.1)	29 (50.9)	1.7747	0.1828			
		T	46	20 (43.5)	26 (56.5)	0.2552	0.6134			
	rs569108	AA	63	28 (44.4)	35 (55.6)		0.653*			
		AG	5	3 (60)	2 (40)					
		GG	0	0 (0)	0 (0)					
		A	-	-	-					
		G	5	3 (60)	2 (40)		0.653*			
C3	rs2230199	CC	2	1 (50)	1 (50)	4.414	0.077	GG	1.75	0.07-43.31
		CG	22	14 (63.6)	8 (36.4)				3.06	1.07-9.22
		GG	44	16 (36.4)	28 (63.6)					
		C	24	15 (62.5)	9 (37.5)	4.2766	0.039	GG	2.91	1.06-8.43
		G	66	30 (45.35)	36 (54.5)		1*			

Gene	SNPs	Genotype	N	Response		χ^2	p-value	Ref Cat	OR	CI 95%
				R<br>N (%)	NR<br>N (%)					
FCGR2A	rs1801274	AA	21	8 (38.1)	13 (61.9)	0.7334	0.693			
		AG	32	16 (50)	16 (50)					
		GG	15	7 (46.7)	8 (53.3)					
		A	53	24 45.3)	29 (54.7)	0.0090	0.924			
		G	21	8 (38.1)	13 (61.9)	0.6877	0.407			
FCGR2B	rs3219018	CC	1	1 (100)	0 (0)	3.0972	0.151			
		CG	21	12 (57.1)	9 (42.9)					
		GG	46	18 (39.1)	28 (60.9)					
		C	22	13 (59.1)	9 (40.9)		0.197*			
		G	67	30 (44.8)	37 (55.2)		0.456*			
	rs1050501	CC	0	0 (0)	0 (0)	0.2223	0.637			
		CT	20	10 (50)	10 (50)					
		TT	48	21 (43.8)	27 (43.8)					
		C	20	10 (50)	10 (50)	0.2223	0.637			
		T	-	-	-					
FCGR3A	rs10127939	AA	62	29 (46.8)	33 (52.2)		0.263*			
		AC	5	1 (20)	4 (80)					
		CC	1	1 (100)	0 (0)					
		A	67	30 (44.8)	37 (55.2)		0.2711*			
		C	62	29 (46.8)	33 (53.2)		0.681*			
	rs396991	AA	25	10 (40)	15 (60)		0.797*			
		CA	34	17 (50)	17 (50)					
		CC	9	4 (44.4)	5 (55.6)					
		A	59	27 (45.8)	32 (54.2)		1*			
		C	25	10 (40)	15 (60)	0.4977	0.481			

Ref. Cat., reference category; R, responder; NR, non-responder; OR, odds ratio; CI 95%, 95% confidence Interval 95%; *p-value for Fisher exact test.
