# Supplementary material for: Influence of Genetics on the Response to Omalizumab in Patients with Severe Uncontrolled Asthma with an Allergic Phenotype
Source: Int J Mol Sci. 2023 Apr 10;24(8):7029. doi: 10.3390/ijms24087029 (PMC10139019; doi:10.3390/ijms24087029)
Supplement: Supplementary file 1 [file ijms-24-07029-s001.zip › Table S3.pdf]

Table S3. Minor allele frequencies for the SNPs studied.

CHR	SNP	Gene	Minor allele	Major allele	MAF
1	rs2427837	FCER1A	A	G	0.1689
1	rs2251746	FCER1A	C	T	0.1892
1	rs1801274	FCGR2A	G	A	0.473
1	rs396991	FCGR3A	C	A	0.3919
1	rs10127939	FCGR3A	C	A	0.0473
1	rs3219018	FCGR2B	C	G	0.1757
1	rs1050501	FCGR2B	C	T	0.1351
2	rs17026974	IL1RL1	A	G	0.2297
2	rs1420101	IL1RL1	T	C	0.3649
2	rs1921622	IL1RL1	A	G	0.4459
3	rs4857855	GATA2	T	C	0.1419
11	rs573790	FCER1B	T	C	0.3243
11	rs1441586	FCER1B	T	C	0.4865
11	rs569108	FCER1B	G	A	0.0473
19	rs2230199	C3	C	G	0.1959
19	rs1054485	FCER1B	T	G	0.4122

Chr, chromosome; SNP, single nucleotide polymorphism; MAF, minor allele frequency.
