# Supplementary material for: Influence of Genetics on the Response to Omalizumab in Patients with Severe Uncontrolled Asthma with an Allergic Phenotype
Source: Int J Mol Sci. 2023 Apr 10;24(8):7029. doi: 10.3390/ijms24087029 (PMC10139019; doi:10.3390/ijms24087029)
Supplement: Supplementary file 1 [file ijms-24-07029-s001.zip › Table S5.pdf]

Table S5. Estimation of haplotype frequency in the reduction and/or absence of oral corticosteroids.

	rs1420101	rs17026974	rs1921622	rs2251746	rs2427837	rs3219018	rs10127939	**Total**	R	NR	Cumulative frequency
1	C	G	G	T	G	G	A	0.3729	0.3527	0.5575	0.3729
2	T	A	A	T	G	G	A	0.1445	0.1208	0.14	0.5174
3	T	G	A	T	G	G	A	0.0975	0.1173	0.02	0.6149
4	C	G	A	T	G	G	A	0.0703	0.0567	0.0625	0.6852
5	C	G	G	C	A	G	A	0.0677	0.0694	NA	0.7529
6	T	A	A	C	A	G	A	0.0435	0.0577	NA	0.7965
7	C	G	G	T	G	C	A	0.0351	0.0291	0	0.8316
8	T	A	A	T	G	C	A	0.032	0.048	0.02	0.8635
9	C	G	A	T	G	C	A	0.0186	0.018	0.02	0.8821
10	C	G	G	C	G	G	A	0.0183	0.0153	NA	0.9004
11	C	G	G	T	G	C	C	0.0139	0.0122	0	0.9143
12	T	G	G	C	A	C	A	0.0135	0.0204	NA	0.9278
13	T	G	A	C	A	C	A	0.0134	0.0198	NA	0.9411
14	C	G	G	C	A	C	C	0.0126	NA	0.0225	0.9537
15	C	G	A	T	G	C	C	0.0083	0.0102	NA	0.962
16	T	G	G	T	G	C	A	0.0076	0.0102	NA	0.9696
17	T	G	G	C	A	G	A	0.0076	0.0111	NA	0.9772
18	C	A	A	T	G	C	C	0.0069	0.0105	NA	0.9841
19	C	G	G	C	A	C	A	0.0049	NA	0.02	0.989
20	C	G	A	C	A	C	C	0.0029	NA	0.0175	0.9919
21	C	G	A	C	A	C	A	0.0029	0.0075	NA	0.9947
22	T	A	A	T	G	C	C	0.0028	NA	0.02	0.9975
23	T	G	A	C	G	G	A	0.002	0.0051	NA	0.9995
24	T	G	A	T	G	C	A	5e-04	NA	0.02	1
25	T	A	A	C	A	C	C	0	0.0079	NA	1

NA, not available; R, responder; NR, non-responder.
