# Supplementary material for: Influence of Genetics on the Response to Omalizumab in Patients with Severe Uncontrolled Asthma with an Allergic Phenotype
Source: Int J Mol Sci. 2023 Apr 10;24(8):7029. doi: 10.3390/ijms24087029 (PMC10139019; doi:10.3390/ijms24087029)
Supplement: Supplementary file 1 [file ijms-24-07029-s001.zip › Table S6.pdf]

Table S6. Estimation of haplotype frequency in lung function improvement.

	rs1420101	rs17026974	rs1921622	rs2251746	rs2427837	rs3219018	rs10127939	**Total**	R	NR	Cumulative frequency
1	C	G	T	G	G	A	0.4605	0.4481	0.5475	0.4605	1
2	T	A	T	G	G	A	0.152	0.1344	0.1712	0.6125	2
3	C	G	C	A	G	A	0.0821	0.0608	0.14	0.6945	3
4	T	G	T	G	G	A	0.0806	0.108	NA	0.7752	4
5	C	G	T	G	C	A	0.0604	0.0751	NA	0.8355	5
6	T	A	C	A	G	A	0.041	0.0371	0.0475	0.8765	6
7	T	A	T	G	C	A	0.0339	0.0378	0.0312	0.9104	7
8	C	G	T	G	C	C	0.0265	0.0307	NA	0.9369	8
9	T	G	C	A	C	A	0.0221	0.0288	NA	0.959	9
10	C	G	C	A	C	C	0.0166	0.0151	NA	0.9755	10
11	C	A	T	G	C	C	0.0084	0.0119	NA	0.984	11
12	T	A	C	G	G	A	0.0074	NA	0.0312	0.9913	12
13	T	G	C	G	G	A	0.0074	0.0096	NA	0.9987	13
14	T	G	T	G	C	A	0.0013	0	NA	1	14
15	T	A	C	A	C	C	0	NA	0.0312	1	15
16	C	G	C	A	C	A	0	0.0025	NA	1	16
17	T	A	T	G	C	C	0	0	NA	1	17

NA, not available; R, responder; NR, non-responder.
