# Supplementary material for: Influence of Genetics on the Response to Omalizumab in Patients with Severe Uncontrolled Asthma with an Allergic Phenotype
Source: Int J Mol Sci. 2023 Apr 10;24(8):7029. doi: 10.3390/ijms24087029 (PMC10139019; doi:10.3390/ijms24087029)
Supplement: Supplementary file 1 [file ijms-24-07029-s001.zip › Table S9.pdf]

Table S9. Estimation of haplotype frequency in the response to the 3 criteria.

	rs1420101	rs17026974	rs1921622	rs2251746	rs2427837	rs3219018	rs10127939	**Total**	R	NR	Cumulative frequency
1	C	G	G	T	G	G	A	0.3895	0.3622	0.5013	0.3895
2	T	A	A	T	G	G	A	0.1516	0.0594	0.1904	0.5411
3	C	G	G	C	A	G	A	0.0846	0.066	0.0539	0.6257
4	T	G	A	T	G	G	A	0.0777	0.129	0.0151	0.7034
5	C	G	A	T	G	G	A	0.0752	0.0945	0.0482	0.7786
6	C	G	G	T	G	C	A	0.0406	0.0395	0	0.8192
7	T	A	A	C	A	G	A	0.0376	0.0469	0.0356	0.8568
8	T	A	A	T	G	C	A	0.0351	0.0711	0.027	0.8919
9	C	G	A	T	G	C	A	0.0158	0.0184	0.0135	0.9076
10	T	G	A	C	A	C	A	0.0147	0.0323	NA	0.9223
11	C	G	G	T	G	C	C	0.0145	0.0161	0	0.9369
12	C	G	G	C	A	C	C	0.0139	NA	0.0258	0.9508
13	C	G	A	T	G	C	C	0.0094	0.0161	NA	0.9602
14	C	A	A	T	G	C	C	0.0074	0.0161	NA	0.9675
15	T	A	A	C	G	G	A	0.0074	NA	0.0135	0.9749
16	T	G	A	C	G	G	A	0.0074	0.0161	NA	0.9822
17	T	G	G	C	A	C	A	0.0074	0.0161	NA	0.9896
18	T	A	A	T	G	C	C	0.0036	NA	0.0151	0.9932
19	T	G	A	T	G	C	A	0.0032	NA	0.0135	0.9964
20	C	G	A	C	A	C	C	0.0026	NA	0.011	0.9991
21	C	G	G	C	A	C	A	9e+04	MA	0.0135	1

NA, not available; R, responder; NR, non-responder.
